# A Practical Roadmap for Implementing the LC-MS-Based Multi-Attribute Method for Analysis of Therapeutic Proteins

**DOI:** 10.3390/ph19101648

**Published:** 2026-10-19

**Authors:** Jingzhong Guo, Sheila Mugabe, Gantasala Subramanya Sameer Kumar, Vishal Amancha, Li Jing, Niomi Peckham, Jarkko Huuskonen, Dipankar Das, Diane McCarthy

**Affiliations:** 1 United States Pharmacopeial Convention, 12601 Twinbrook Parkway, Rockville, MD 20852, USA; tim.guo@usp.org (J.G.); li.jing@usp.org (L.J.); niomi.peckham@usp.org (N.P.);; 2 United States Pharmacopeia India Pvt Ltd., Hyderabad 500101, Telangana, India

**Keywords:** multi-attribute method (MAM), LC-MS, peptide mapping, biopharmaceutical quality control, method qualification, system readiness, assay transfer, critical quality attribute (CQA) monitoring

## Abstract

**Background/Objectives:** The mass spectrometry-based multi-attribute method (MAM) enables site-specific monitoring of multiple product quality attributes (PQAs) within a single LC-MS peptide mapping assay and is increasingly being adopted in biopharmaceutical development and quality control (QC). However, its analytical and operational complexities necessitate a structured approach to method development, qualification, system readiness, and assay transfer. This study presents a practical roadmap for implementing LC-MS-based MAM, aligned with USP-NF General Chapter 〈1060〉. **Methods:** Sample preparation and LC-MS acquisition parameters were optimized to minimize analytical artifacts and support robust workflow performance. System readiness was established using a publicly available monoclonal antibody reference standard. Adalimumab and etanercept were then used as representative biotherapeutics to evaluate key elements of MAM implementation, including qualification and interlaboratory transfer. **Results:** The refined workflow provided consistent digestion performance, chromatographic stability, mass accuracy, and reliable data processing across both molecules. Qualification studies demonstrated acceptable specificity, precision, sensitivity, and quantitative performance for targeted PQA monitoring, while new peak detection (NPD) assessments supported the detection of emerging or changing species. Interlaboratory transfer studies demonstrated successful transfer of the standardized workflow across laboratories and LC-MS platforms. **Conclusions:** These results establish a technical and operational foundation for MAM implementation, including system readiness, qualification, and assay transfer. The proposed implementation framework provides a robust and broadly applicable roadmap for MAM deployment across diverse biopharmaceutical programs while supporting subsequent product-specific refinement and validation for routine QC applications.

## 1. Introduction

The structural and functional complexity of monoclonal antibodies (mAbs) and related biotherapeutic proteins continues to challenge traditional analytical control strategies in quality control (QC) laboratories [1,2,3,4]. Modern biologics have numerous product quality attributes (PQAs) including post-translational modifications (PTMs) [5,6,7,8,9,10], sequence variants [11], size variants [12], charge variants [11], and structural heterogeneity [13] that can affect product safety, efficacy, and stability. The need to employ numerous analytical methods to assess diverse product attributes has increased interest in more efficient approaches that enable simultaneous monitoring of multiple attributes with the specificity, sensitivity, and robustness required for routine QC deployment [14].

Among these, the mass spectrometry-based multi-attribute method (MAM), most commonly implemented via peptide mapping LC-MS, has emerged as a central platform for characterization and routine monitoring of PQAs in therapeutic proteins [15,16,17,18,19,20,21,22,23,24,25]. By leveraging high-resolution accurate mass spectrometry (HRAM) and targeted peptide-level analysis, MAM supports simultaneous identification and relative quantitation of multiple PQAs, including charge heterogeneity, N and C-terminal variants, glycosylation profiles, cysteine-related modifications (e.g., disulfide scrambling, free thiols), oxidation, deamidation, and isomerization, within a single assay [15,16,17,18,19,22,26,27,28,29]. This multiplexed, direct measurement of PQAs provides a more information-rich and streamlined alternative to traditional orthogonal assay panels, enabling deeper molecular characterization and supporting enhanced understanding of both the manufacturing process and the product throughout its lifecycle.

Recent advances have strengthened MAM’s suitability for broader applications beyond R&D-related analytical characterization and have increased interest in its potential use in regulated laboratory environments, including QC settings [30,31,32,33,34]. Improvements in MS instrumentation and chromatographic robustness [29,35], the adoption of automated sample preparation [36], advances in NPD algorithms [24,29], and more sophisticated informatics tools have collectively enhanced the method’s ability to deliver site-specific PTM quantitation, detect low-level sequence variants, and identify both targeted and nontargeted emerging product-related species within a single workflow [33]. In parallel, industry efforts have demonstrated the successful implementation of targeted MAM approaches using both high-resolution and low-resolution mass spectrometry platforms for identification testing, attribute monitoring, and QC applications [15,20,33,37,38]. Together, these advances position MAM as a highly informative and potentially more efficient alternative to traditional assay panels for release and stability testing in QC environments.

Despite these benefits, several practical and regulatory challenges have limited broader adoption of MAM in QC environments [22,34]. Key considerations include risk assessment, method qualification and validation, evaluation of NPD capability and specificity, and performance comparisons with conventional methods. Additional needs include clearly defined system suitability criteria [33]; strategies to minimize artifacts induced by sample preparation [16,28,29,39,40]; management of missed cleavages, chromatographic tailing, and sample carryover; robust and standardized instrument settings, data-processing parameters, and software workflows capable of supporting targeted PQA monitoring, NPD evaluation, and data review in regulated environments [22,24,27]; long-term instrument control strategies; and guidance to maintain method performance across sites and throughout the product lifecycle. Addressing these issues requires structured, consensus-based guidance to support consistent implementation and facilitate broader adoption following appropriate product-specific qualification and validation activities. In addition, product-specific assessments of specificity remain necessary to support routine QC implementation because analytical performance may vary depending on molecular structure, formulation components, and relevant degradation pathways.

Stakeholders have increasingly recognized MAM as a priority area for analytical standardization. In 2020, attendees at a United States Pharmacopeia (USP) Stakeholder Forum on Biologics identified the need for both documentary standards and physical reference materials to support consistent implementation [41]. In response, USP convened an Expert Panel of industry scientists and regulatory representatives to draft a general chapter on MAM.

General Chapter 〈1060〉 provides industry with the best practices and considerations to guide the development, qualification, implementation, and lifecycle oversight of MAM workflows [42]. The chapter promotes a platform-based framework that can be adapted to different biotherapeutic products while recognizing that product-specific optimization, qualification, critical quality attribute (CQA) selection, and acceptance criteria remain necessary for individual applications. This chapter also offers practical recommendations on method design, sample preparation, LC-MS system configuration, software requirements, instrument readiness, system suitability, targeted PQA monitoring, and nontargeted analysis (including reduced and nonreduced peptide mapping platforms), while retaining the flexibility required for diverse products and platforms. The chapter became official in August 2025, providing a comprehensive framework to enable broader and more standardized adoption of MAM in QC laboratories without imposing compendial obligations.

The present work, conducted in part with support from the U.S. Food and Drug Administration (FDA), contributes to the evaluation of MAM as an analytical approach to support biotherapeutic development and QC. The overall study design included acquisition of adalimumab and etanercept from multiple sources and the application of thermal and chemical stress conditions to generate a range of product modifications suitable for assessing MAM performance. Within this broader context, this article focuses on key elements for implementing MAM, including the development of a broadly applicable workflow, system readiness assessment, method qualification, and interlaboratory transfer using a limited set of adalimumab and etanercept samples. Applications to the full set of unstressed and stressed samples, along with comprehensive comparisons to conventional orthogonal methods, are out of scope of the present study and will be presented in a subsequent article.

This article provides a practical framework and roadmap for implementing MAM, emphasizing workflow elements that are broadly transferable across biotherapeutic products, including system readiness assessment, method qualification, interlaboratory transfer, and lifecycle management principles. Adalimumab and etanercept are used as representative products to illustrate key principles and practices across characterization, targeted PQA monitoring, NPD evaluation, method qualification, and interlaboratory transfer. The primary contribution of this work is the systematic integration of these established MAM elements into a structured implementation framework that supports MAM deployment in QC environments.

## 2. Results

The results presented in this section are organized to follow the MAM lifecycle outlined in General Chapter 〈1060〉, progressing from framework definition through method development, qualification, system readiness evaluation, and ultimately interlaboratory performance assessment.

### 2.1. Overview of Approach to Development of a Roadmap for MAM Implementation

**Figure 1:**
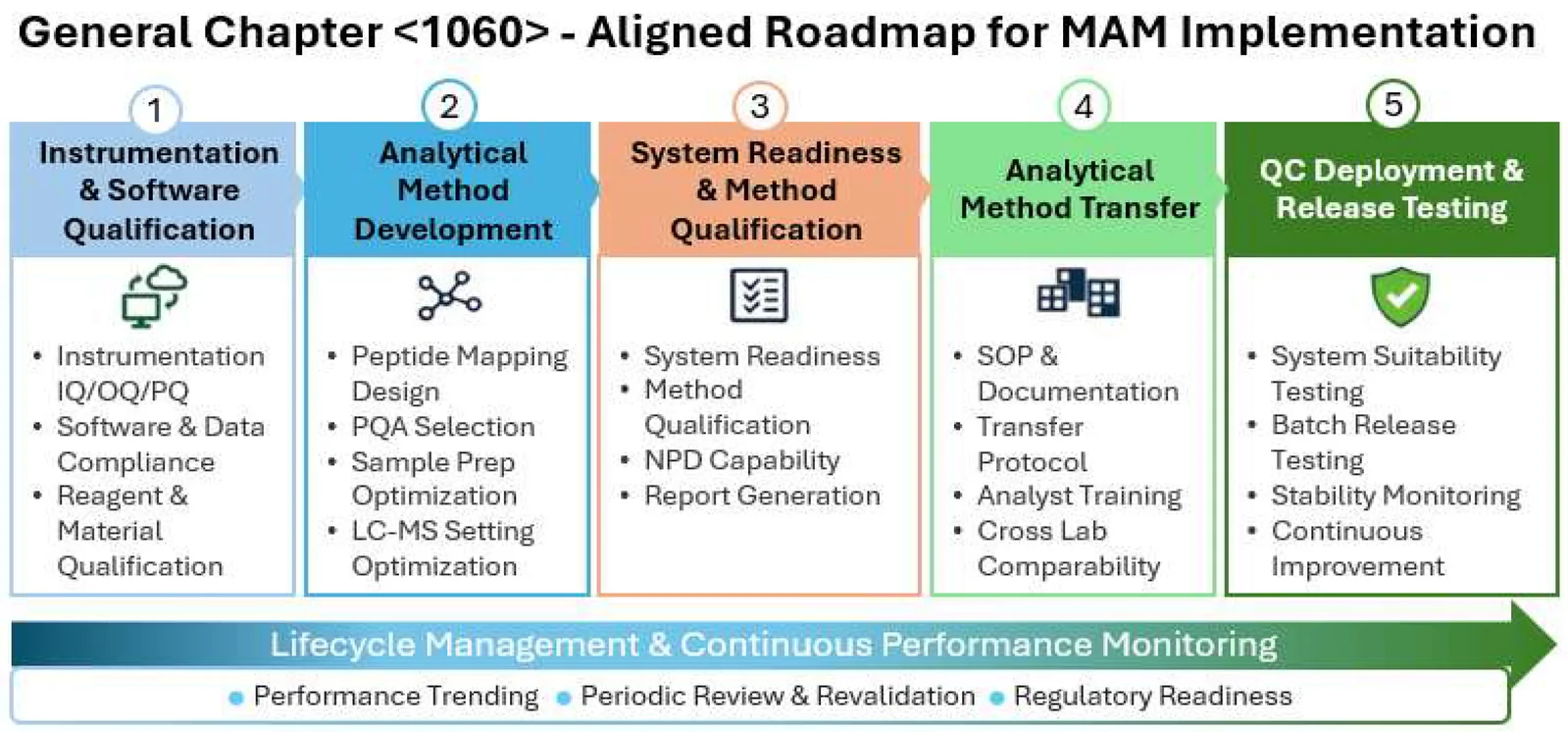
Conceptual Roadmap for MAM Implementation Aligned with General Chapter 〈1060〉. This figure was created using Microsoft^®^ PowerPoint^®^ for Microsoft 365 MSO (Version 2608). Certain graphical elements and illustrations in this figure were generated using Microsoft Copilot accessed via Microsoft Teams (Version 26260.1701.5139.3736).

MAM is an integrated analytical system in which sample preparation, LC-MS instrumentation, data-processing software, and system-readiness controls work together to enable reliable, attribute-specific monitoring of therapeutic proteins. This integrated framework provides the foundation for a practical roadmap for MAM implementation in biopharmaceutical QC laboratories, as illustrated in Figure 1. Although instrument qualification and overarching lifecycle management are integral to a complete MAM workflow, they are not addressed in detail here. This article focuses on the core stages that support progression toward regulated and QC-oriented use: sample preparation and method optimization, system readiness evaluation, attribute-centered method qualification, and assay transfer across laboratories or LC-MS platforms. These four elements of the MAM roadmap lay the groundwork for routine QC implementation and ongoing lifecycle oversight. Through representative case studies, the roadmap illustrates how these sequential elements can be practically integrated into a structured implementation framework that can be adapted to different biotherapeutic products. Product-specific optimization, qualification, attribute selection, and acceptance criteria remain necessary depending on the molecule and intended application.

A QC-ready roadmap begins with robust, low-artifact sample preparation. Digestion conditions, pH control, enzyme selection, and mitigation of method-induced modifications (such as deamidation or oxidation) are carefully optimized and controlled to ensure reproducibility across drug substances (DS), drug products (DP), stability and in-process samples, thereby supporting subsequent method qualification and future assay transfer.

Together with controlled sample preparation, General Chapter 〈1060〉 highlights system readiness as a central element of MAM implementation. System readiness provides real-time confirmation that the end-to-end MAM workflow, from sample preparation through LC-MS acquisition and automated data processing, remains within predefined acceptance criteria. Typical metrics include retention time stability, signal intensity, mass accuracy, resolution, and levels of artifact PTMs, often assessed using a suitable reference standard. Embedding system readiness checks before and during analytical runs ensures the MAM system remains fit for use at the time of data acquisition. Successful implementation also depends on software platforms capable of supporting targeted attribute monitoring, NPD evaluation, data review, and compliant data management, although the concepts described here are not restricted to a specific software package. General Chapter 〈1060〉 distinguishes between system readiness and system suitability to address different risks at different stages of the MAM lifecycle. System readiness verifies that the integrated MAM system is capable of reliable data generation, while system suitability formally demonstrates—at the time of testing—that the validated method meets predefined performance criteria for reportable, cGMP-compliant results.

With sample preparation and system readiness established, the next step is fit-for-purpose method qualification, including evaluation of repeatability, intermediate precision, robustness, specificity, and NPD capability under conditions relevant to routine use. In this work, these assessments were conducted within a qualification framework to characterize method capability and variability at the attribute level. While such qualification establishes confidence in analytical performance and supports downstream method development, full method validation remains a necessary subsequent step for final QC implementation, ensuring reliable routine monitoring of targeted attributes and robust detection of unexpected changes.

Following method qualification, the roadmap progresses to assay transfer across laboratories and/or LC-MS platforms, a key step toward broader, QC-oriented implementation. General Chapter 〈1060〉 emphasizes the use of harmonized standard operating procedures (SOPs), locked data-processing methods, predefined acceptance criteria, and system readiness benchmarks to promote method consistency across sites. In this framework, interlaboratory comparability is assessed at the attribute level, consistent with the QC-relevant control principles, rather than through absolute matching of instrument response.

The final stage of the roadmap addresses routine use in regulated environments, representing a pathway toward formal QC deployment. Ongoing trending of system readiness metrics, management of changes to instruments, reagents, and software, and periodic review of system suitability performance are critical to sustain method performance over time and to support continued control of monitored critical quality attributes throughout the product lifecycle.

### 2.2. MAM Method Development Using Adalimumab and Etanercept

Building on the framework described above, method development efforts were focused on establishing a robust and transferable MAM workflow.

In this study, adalimumab (an IgG1 mAb) and etanercept (a TNF receptor-Fc fusion protein) were selected as representative molecules based on their well-documented structures and established PTM profiles. Together, these products represent common classes of therapeutic proteins and were selected as representative case studies to demonstrate the implementation framework across molecules with differing structural features and PTM profiles. MAM method development within this study leveraged prior experience with LC-MS peptide-mapping workflows and focused on implementation and refinement of established MAM principles. Emphasis was placed on controlled sample preparation, attribute-informed peptide selection, and early locking of LC-MS acquisition and data-processing parameters to support subsequent assay qualification and transfer. Parameters with the greatest potential impact on reproducibility and attribute stability were selected for detailed evaluation.

#### 2.2.1. Attribute-Informed Peptide Selection

Attributes monitored by MAM were selected based on established critical quality attributes and known structure-function relationships for adalimumab and etanercept. Representative attributes and their corresponding peptide regions are summarized in Table 1 (see Supplemental Tables S1 and S2 for selected quality attributes for adalimumab and etanercept).

In addition to methionine oxidation and asparagine deamidation—which were included due to their known effects on conformational stability, potency, and charge heterogeneity [7,43,44,45]—the MAM panel encompassed several additional PQAs. N-terminal pyroglutamate formation was monitored because of its influence on charge variants and N-terminal integrity [11,46], while C-terminal lysine clipping was tracked due to its contribution to charge heterogeneity and its relevance to lot comparability [11,25,47]. Proline amidation has been reported to impact basic-charged variant levels [48], while Asp isomerization within the CDR regions can decrease binding affinity and potency [49]; lysine glycation was evaluated due to its potential to increase aggregation during long-term storage, at higher temperature or lower pH [50]. Cleavage at Asp-Pro motifs (DP clipping) was included because it affects fragmentation susceptibility, a key stability-related critical quality attribute [51]. Ser/Thr O-glycosylation was monitored due to its contributions to size and charge heterogeneity, particularly for etanercept, where O-linked glycans represent a substantial portion of total glycan mass [8,52]. N-glycosylation in the Fc domain was assessed because of its established impact on effector function-related properties such as FcγR binding and ADCC potential [8,53,54,55,56].

Overall sequence coverage was tracked to support identity confirmation and to provide additional context for interpreting attribute-specific results. Although the achieved sequence coverage was sufficient to support the objectives of this study, regions with incomplete coverage may reduce the ability to monitor certain modifications or unexpected species and should be evaluated on a product-specific basis. Where coverage of relevant PQAs is insufficient, alternative digestion strategies may be required to achieve adequate monitoring of those attributes. Only peptides demonstrating robust detectability and stable chromatographic behavior under optimized MAM conditions were advanced to qualification.

#### 2.2.2. Sample-Preparation Optimization

Sample-preparation parameters were evaluated to ensure consistent digestion performance while minimizing artifactual modifications. Based on prior internal studies and literature demonstrating the impact of sample preparation on PTM integrity and digestion efficiency, denaturation, reduction/alkylation, digestion, and cleanup conditions were selected for assessment within QC-relevant ranges. Parameters that were evaluated are summarized in Table 2 to illustrate the considerations used in establishing an optimized workflow. Denaturation and reduction/alkylation conditions were chosen to achieve efficient protein unfolding and disulfide bond reduction while minimizing oxidation and disulfide scrambling, as these factors have been observed to be sensitive to reagent concentration and incubation conditions. Digestion time was evaluated over a limited range based on prior findings that extended digestion can improve sequence coverage but may also increase artifactual modifications, requiring a balance between coverage and PTM stability. In addition, different commercially available trypsin grades were evaluated to assess their impact on digestion efficiency and variability; based on these comparisons, a consistent, high-quality trypsin grade was selected for routine use to minimize enzymatic variability.

Different quality attributes exhibited variable sensitivity to changes in sample-preparation conditions. Certain PTMs—including N-glycosylation, DP clipping, C-terminal Lys clipping, and N-terminal pyroglutamate formation—showed minimal or negligible variation across conditions. In contrast, methionine oxidation, asparagine deamidation, and/or succinimide intermediate formation were markedly influenced by denaturation and digestion parameters. Supplemental Figure S1 compares the relative abundance of representative adalimumab quality attributes under six sample-preparation conditions.

Across these conditions, higher denaturation temperatures and extended digestion times consistently increased methionine oxidation, deamidation, and succinimide formation. Under equivalent conditions, a high-purity, platinum MS-grade trypsin produced lower levels of these PTMs and reduced trypsin autolysis compared with a standard sequencing grade trypsin. Similar trends were observed for etanercept.

**Figure 2:**
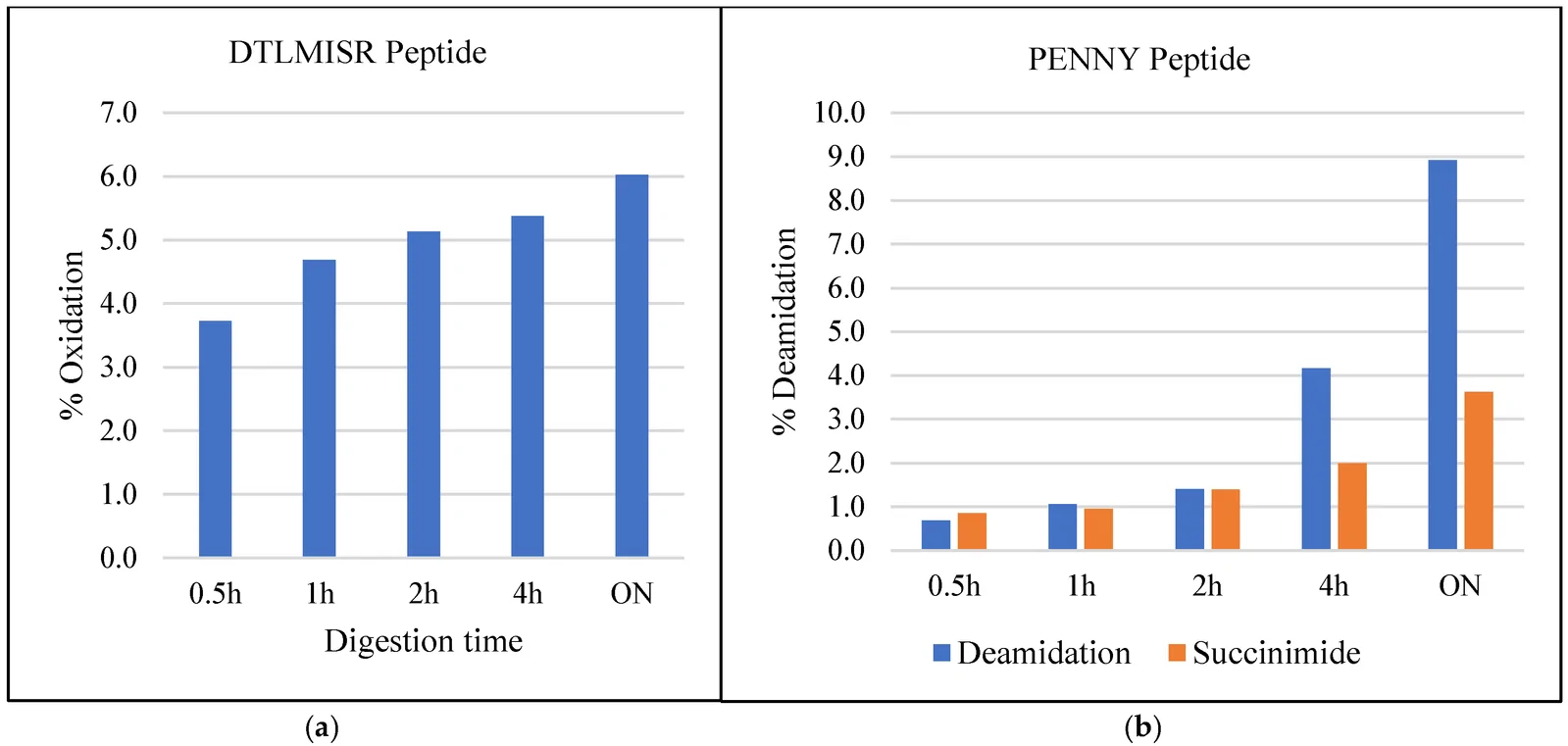
Assessment of trends associated with digestion time, including (**a**) oxidation of DTLMISR peptide, (**b**) deamidation and succinimide formation in the PENNY peptide, both derived from etanercept. ON represents overnight incubation (18 h).

Figure 2 presents a time-course analysis of oxidation of the DTLMISR peptide and oxidation and deamidation/succinimide formation of the etanercept PENNY peptide (referring to the peptide GFYPSDIAVEWESNGQPENNYK containing the characteristic ‘PENNY’ residue sequence motif), selected due to its susceptibility to multiple PTMs and sensitivity to digestion conditions. All three PTMs increased with longer digestion times. Deamidation and succinimide formation were largely minimized when digestion was restricted to under 2 h; however, methionine oxidation continued to rise markedly between 0.5, 1, and 2 h. Similar time-dependent trends were observed across other Met-containing and Asn-containing peptides in both adalimumab and etanercept, supporting the general impact of digestion time on oxidation and deamidation-related modifications.

**Figure 3:**
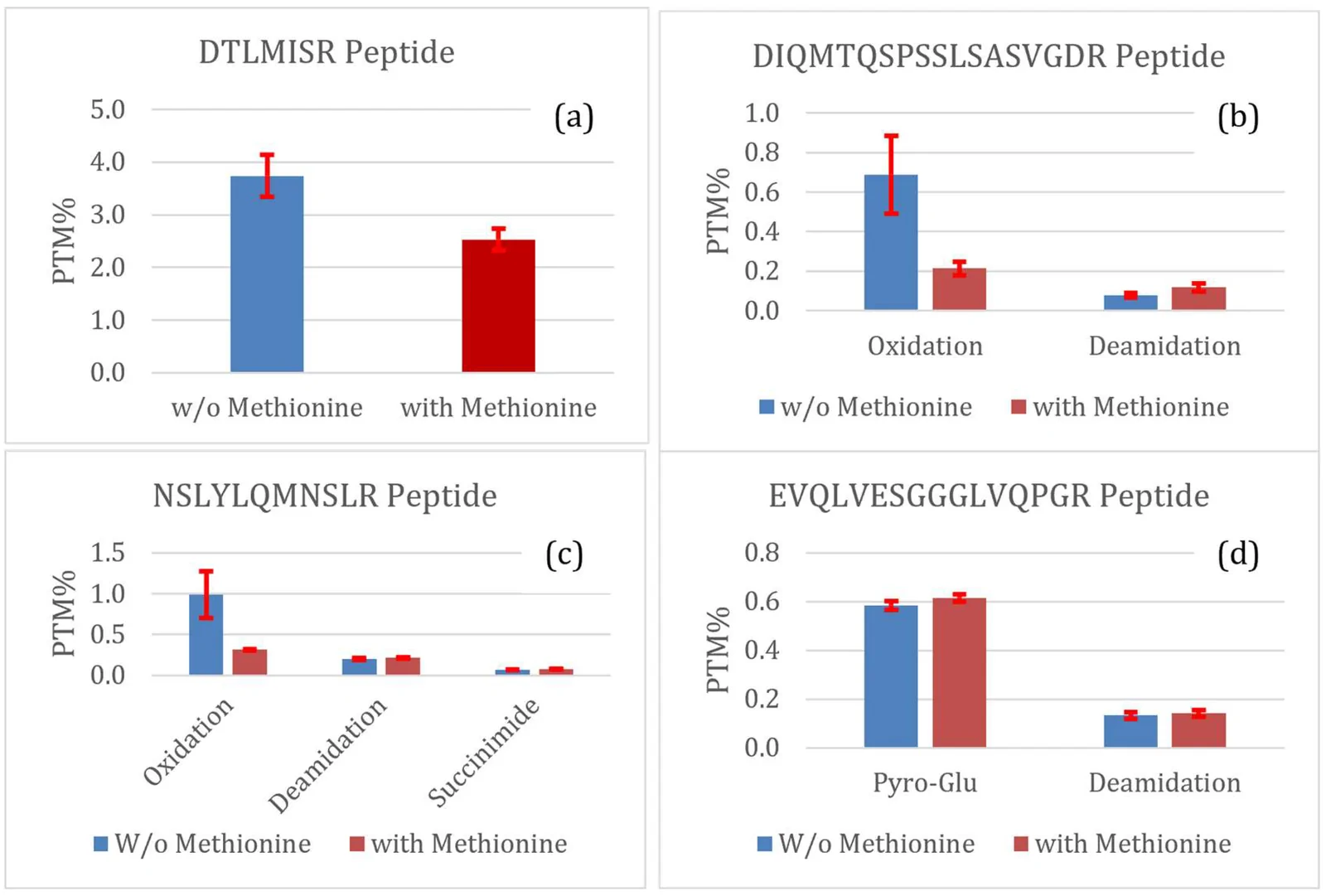
Comparison of PTM levels in representative adalimumab peptides prepared with and without 10 mM methionine: (**a**) oxidation of DTLMISR peptide; (**b**) oxidation/deamidation of DIQMTQSPSSLSASVGDR peptide; (**c**) oxidation/deamidation/succinimide of NSLYLQMNSLR peptide; and (**d**) pyroglutamate/deamidation of EVQLVESGGGLVQPGR peptide. Error bars represent ±1 standard deviation based on two injections from each of three independent sample preparations (*n* = 6 total injections).

To reduce sample preparation-induced oxidation, 10 mM methionine was incorporated into the denaturation and digestion buffers. As shown in Figure 3, methionine supplementation effectively decreased oxidation in methionine-containing peptides without substantially affecting other PTMs. Additionally, the presence of methionine significantly reduced variability in oxidation measurements, yielding tighter standard deviations across replicate preparations and injections.

In summary, artifactual PTMs were minimized by limiting digestion to under 2 h, using a platinum MS-grade trypsin, and including 10 mM methionine in the denaturation and digestion buffers. This combination reduced methionine oxidation and improved reproducibility without impacting other monitored PTMs. The final sample preparation conditions are described in detail in Section 4.2.

#### 2.2.3. Optimization of LC-MS Conditions

**Figure 4:**
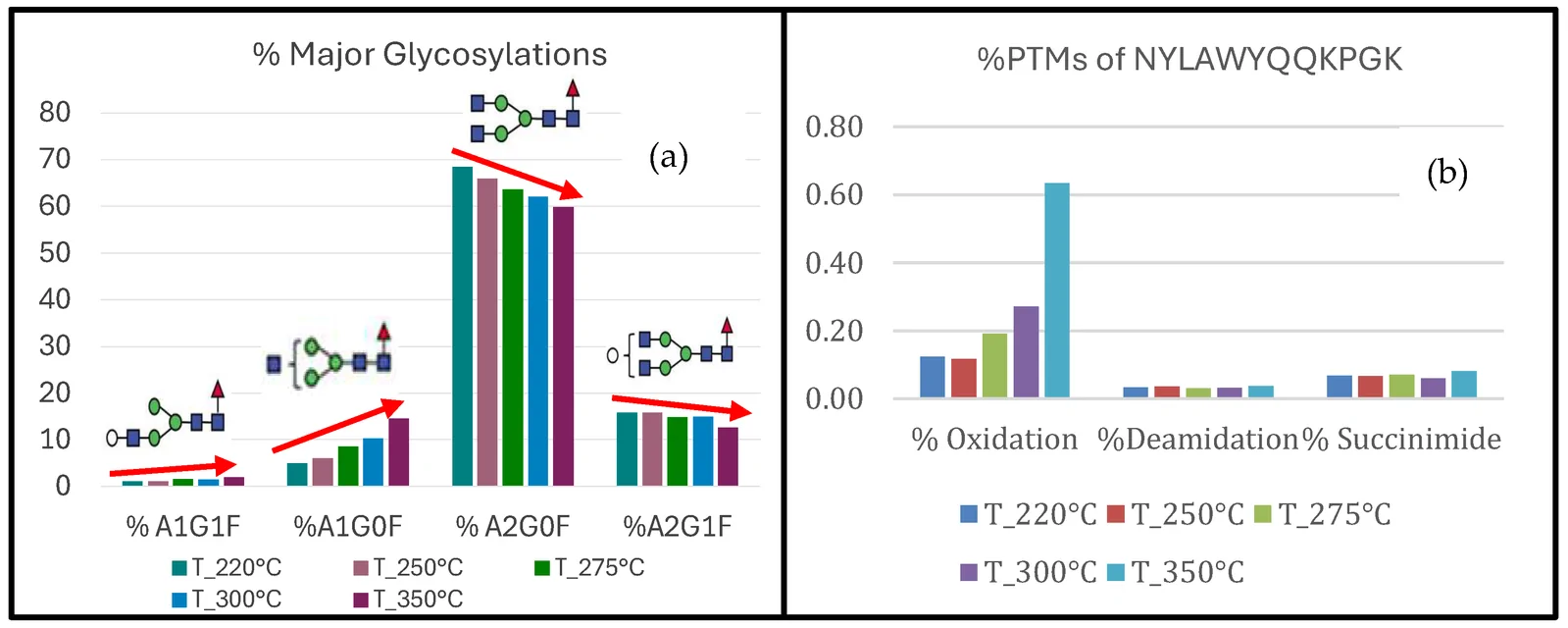
Trend in relative abundance as a function of ion-source temperature (220–350 °C) for (**a**) major N-glycosylation species and (**b**) oxidation, deamidation, and succinimide formation in adalimumab. Red arrows indicate the direction of glycan abundance changes with increasing temperature. Glycan structures are shown using the Symbol Nomenclature for Glycans (SNFG), where blue squares denote N-acetylglucosamine (GlcNAc), green circles denote mannose (Man), yellow circles denote galactose (Gal), and red triangles denote fucose (Fuc).

Commonly used and manufacturer-recommended LC-MS acquisition settings and data-processing workflows were used as a starting point to minimize variability and support reproducible performance. From this baseline, minor variations in acquisition parameters, including source voltage, sheath gas, cycle time, RF lens settings, mass resolution, and collision energy, slightly affected MS response but had negligible impact on the relative quantitation of %PTMs. In contrast, ion source temperature was evaluated following optimization of sample-preparation conditions and was found to significantly influence measured %glycosylation, while having minimal impact on other PTMs. Figure 4a illustrates the trend in relative abundance of major N-glycosylation species and other PTMs of adalimumab as a function of ion source temperature (220–350 °C). Increasing ion source temperature promoted cleavage of terminal antenna GlcNAc residues, resulting in decreased A2G0F and A2G1F and corresponding increases in A1G0F and A1G1F. Other PTMs in adalimumab were largely insensitive to temperature changes, except for oxidation, which increased at temperatures above 250 °C (Figure 4b). A similar trend was observed for PTM measurements in etanercept. Based on this evaluation, an ion source temperature of 225 °C was selected for the final method, as it significantly reduced the apparent decomposition of highly branched glycans without compromising MS response.

### 2.3. System Readiness Evaluation

System readiness was evaluated in accordance with General Chapter 〈1060〉 principles and focused on chromatographic stability, mass-spectrometric performance, and the reproducibility of measurements for sensitive product attributes. A publicly available mAb standard, USP mAb 001 Reference Standard (RS), was used as a system readiness standard. USP mAb 001 RS is a well-characterized IgG1 mAb reference material with attributes and PTMs typical of most mAbs, making it well suited for establishing system readiness and assessing the end-to-end performance of MAM workflows. Seven peptides from USP mAb 001 RS were selected to evaluate system readiness. The system readiness metrics included 17 parameters, encompassing total ion chromatogram MS intensity, mass accuracy, MS peak intensity and retention time of individual peptides, and %oxidation of the DTLMISR peptide. Supplemental Figure S2 presents the extracted ion chromatograms of the seven peptides spanning the full LC gradient, demonstrating consistent peptide detection across early-, mid-, and late-eluting regions. Supplemental Table S3 provides representative results for all 17 system readiness parameters from a single run.

Across all evaluated peptides, mass accuracy remained within the predefined acceptance limit (≤5 ppm), confirming stable instrument calibration throughout the analytical sequence. Signal response and peak-area precision also met predefined criteria (total ion chromatogram intensity within the expected range and peptide peak areas ≥1 × 10^6^), indicating adequate MS sensitivity and injection-to-injection reproducibility.

**Figure 5:**
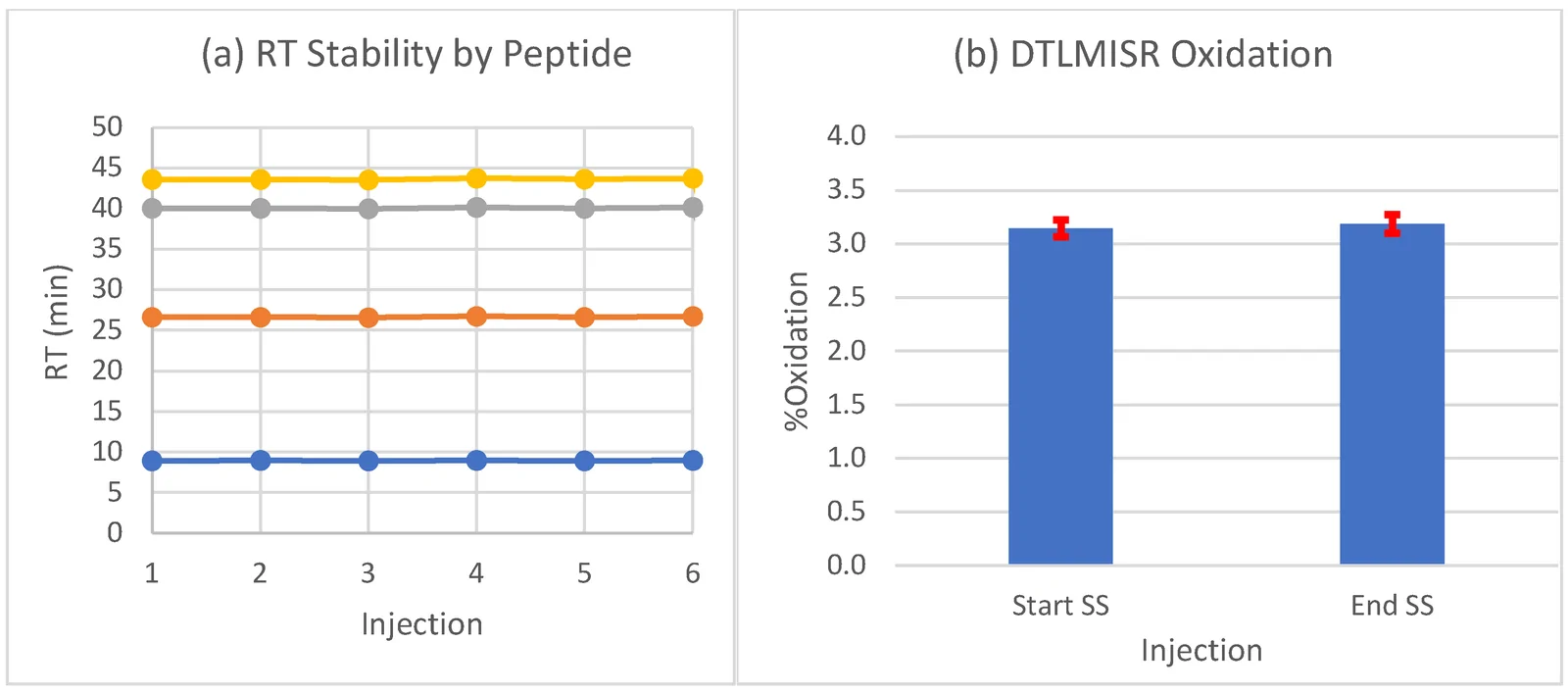
Representative system performance using USP mAb 001 RS: (**a**) Injection-to-injection retention time stability for core peptides: VDNALQSGNSQESVTEQDSK (blue), TPEVTC[Carbamidomethylation]VVVDVSHEDPEVK (orange), TTPPVLDSDGSFFLYSK (grey) and TVAAPSVFIFPPSDEQLK (yellow); (**b**) DTLMISR %oxidation measured at the beginning and end of analytical sequences (>72 h per run). Bars represent the average %oxidation across three independent runs conducted in different weeks; error bars denote ±1 standard deviation, reflecting inter-run variability.

Figure 5 illustrates representative system performance across multiple runs using selected parameters. Figure 5a shows injection-to-injection retention-time stability for four representative peptides: VDNALQSGNSQESVTEQDSK (blue), TPEVTC[carbamidomethylation]VVVDVSHEDPEVK (orange), TTPPVLDSDGSFFLYSK (grey), and TVAAPSVFIFPPSDEQLK (yellow). The low retention-time variability observed across replicate injections (%RSD = 0.14–0.27%) demonstrates robust and reproducible chromatographic performance suitable for quantitative MAM applications.

To further assess the system’s ability to reliably quantify labile PTMs, methionine oxidation was monitored using the oxidation-prone peptide DTLMISR. Oxidation levels were compared at the beginning and end of each analytical sequence to assess intra-sequence stability over extended acquisition periods (>72 h per run). As shown in Figure 5b, the bar plot presents the average %oxidation at start and end of the sequences across three independent runs conducted over different weeks. The consistently low oxidation levels (~3%), with tight variability (%RSD ≈ 2.5%), as reflected by the small error bars, indicate robust and reproducible PTM measurements with no evidence of systematic drift over the course of prolonged analyses.

Collectively, these observations indicate that the LC-MS system consistently met predefined readiness criteria for chromatographic stability, mass accuracy, signal precision, and PTM monitoring. The resulting data confirm that the analytical system was suitable for MAM analysis and that product-specific results generated during these sequences were supported by verified system performance.

### 2.4. MAM Assay Qualification

Assay qualification and assay validation represent different stages of method maturity. For QC applications, full validation is ultimately required and considered part of the overall implementation. Moreover, method validation is typically performed on a specific therapeutic protein once the set of reportable attributes has been defined. In this study, our objective was to qualify a MAM workflow that can serve as a starting point for a broad range of mAbs and other therapeutic proteins, while recognizing that product-specific attribute selection, optimization, qualification, and validation remain necessary for individual molecules and applications. For some performance characteristics, including accuracy, linearity, detection limit, and quantitation limit, representative peptides/PTM pairs were used to illustrate the qualification approach. Because individual peptides and PTMs may differ in recovery, chromatographic behavior, ionization efficiency, chemical stability, signal response, and susceptibility to matrix interference, the results obtained for these representative analytes should not be assumed to apply to all monitored PQAs. Additional attribute-specific evaluation may therefore be appropriate depending on the physicochemical characteristics, expected range, and intended use of each monitored attribute. These results demonstrate workflow performance for selected model analytes and may not necessarily reflect the behavior of all monitored PQAs. Method qualification for MAM was conducted in alignment with ICH Q2(R2) requirements [57,58] for analytical procedure validation and *USP–NF* General Chapter 〈1225〉 Validation of Compendial Procedures [42,59]. The qualification approach followed the General Chapter 〈1060〉 framework, which emphasizes the integration of sample preparation, LC-MS instrumentation, and MAM analysis software, all of which collectively determine method suitability. Qualification activities focused on the specificity of attribute monitoring, repeatability and intermediate precision, linearity and range, and robustness to small, deliberate method perturbations. In addition, NPD capability was evaluated as part of the overall control strategy.

#### 2.4.1. Specificity

Specificity for this platform-method qualification was assessed in line with ICH guidelines, which define specificity as the ability to assess unequivocally the analyte in the presence of components that may be expected to be present, such as impurities, degradation products, and matrix components. Consistent with this, targeted peptides and their PTMs were uniquely identified based on accurate mass, retention time, and isotopic pattern, with MS/MS support during method development.

**Figure 6:**
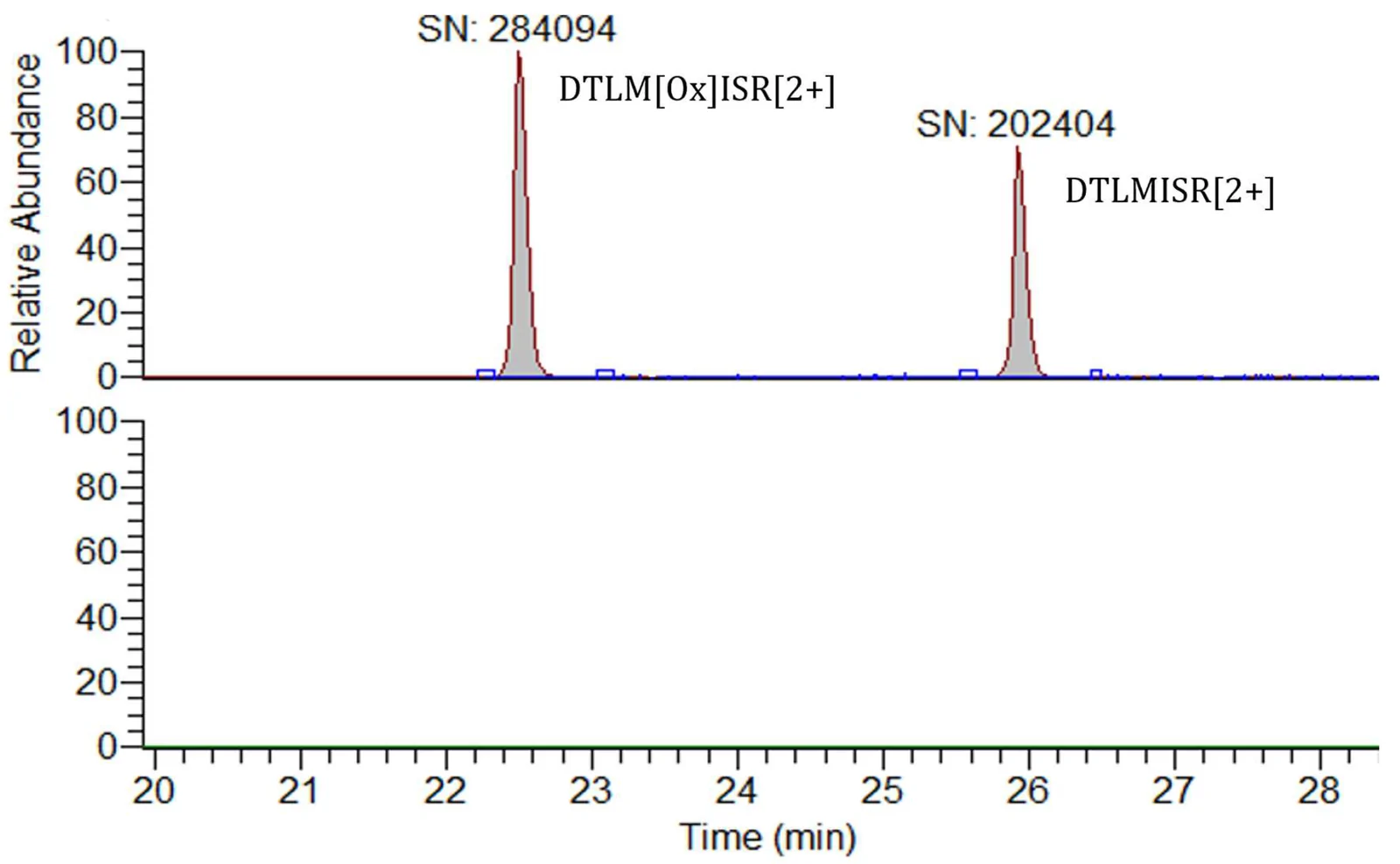
Extracted Ion Chromatogram of DTLM[Ox]ISR [^2+^] (*m*/*z* = 426.2182, 5 ppm) and DTLMISR [^2+^] (*m*/*z* = 418.2208, 5 ppm) from adalimumab sample (**top**) vs. Blank sample (**bottom**). This figure was generated using Xcalibur software (version 4.7.102.25, Thermo Fisher Scientific, Waltham, MA, USA).

Specificity was evaluated using formulation blanks and cross-protein comparison between adalimumab and etanercept as an unrelated protein matrix. Shared peptides (e.g., conserved Fc-region peptides and EEQYNSTYR) were consistently detected in both digests, while protein-specific peptides (e.g., etanercept O-glycopeptides) were absent in the alternate digest. Across the monitoring panels (15 peptides for adalimumab and 20 for etanercept), no interfering peaks were observed within predefined extraction criteria in blanks or alternate-protein digests. Figure 6 illustrates this using DTLMISR peptide and its oxidized form from the adalimumab sample, showing clear discrimination from background in the blank.

These results support qualification of the workflow with respect to specificity by demonstrating the ability to distinguish monitored analytes from formulation components and unrelated protein matrices under the evaluated conditions. Additional evaluation of relevant product-related variants, impurities, degradation products, stressed samples, and product-specific matrix effects would be required during product-specific validation before routine QC implementation.

#### 2.4.2. Accuracy

Accuracy was evaluated using spike-and-recovery testing of multilevel mixtures in line with General Chapter 〈1060〉 recommendations for quantitative assessment of PQAs. In this study, accuracy was estimated based on recovery results from the linearity experiments using two representative peptide pairs, DTLMISR and DIQMTQSPSSLSASVGDR, together with their corresponding oxidized forms. For both native and oxidized DTLMISR, recoveries were within 0.4–10% of the nominal amount over the testing range (0–3 µg protein). Similar results were obtained for native and oxidized DIQMTQSPSSLSASVGDR, with recoveries within 0.8–10% of the nominal amount. These results demonstrate acceptable quantitative performance across representative peptides with different sequences and chromatographic characteristics. This representative peptide approach was used to evaluate the overall capability of the platform workflow.

#### 2.4.3. Precision

Repeatability and intermediate precision were evaluated across analysts, instruments, and days. Repeatability was assessed by analyzing replicate injections from independently prepared digests under identical analytical conditions. Intermediate precision was examined through multi-day analyses designed to capture variability encountered during routine QC operation, including, where applicable, different analytical days and instrument sessions. Precision assessments considered retention time consistency, peak area repeatability, and the relative abundance of selected PTM attributes. As noted in General Chapter 〈1060〉, precision may vary among peptides and attributes depending on their chemical lability (e.g., oxidation, deamidation) and susceptibility to digestion-related artifacts, and this behavior was observed during qualification. Supplemental Table S4 summarizes the repeatability results for adalimumab PQAs based on six injections of three independently prepared sample replicates. Supplemental Table S5 presents precision (*n* = 6) and intermediate precision (*n* = 12) results for etanercept PQAs. Most attributes showed good precision (RSD < 8%). Slightly higher variability (RSD ~10%) was observed for two PQAs in adalimumab and one PQA in etanercept, with VSNK showing an RSD of approximately 10% in both products. The higher variability may be associated with their relatively low MS signal responses. Overall, the method demonstrated consistent precision for all targeted attributes within the established range.

#### 2.4.4. Linearity and Range

Linearity was assessed using sample mixtures prepared by combining materials with defined and contrasting levels of the target attribute, consistent with the attribute-based method evaluation principles outlined in General Chapter 〈1060〉. For adalimumab, the control material exhibited only trace levels of oxidation, whereas the hydrogen peroxide-treated material contained near-complete oxidation at the selected methionine sites. Binary mixtures of the control and oxidized materials were prepared at control-to-oxidized ratios of 0:1, 1:3, 1:2, 1:1, 2:1, 3:1, and 1:0. For each mixture, 3 µg of total protein was injected for LC-MS analysis.

**Figure 7:**
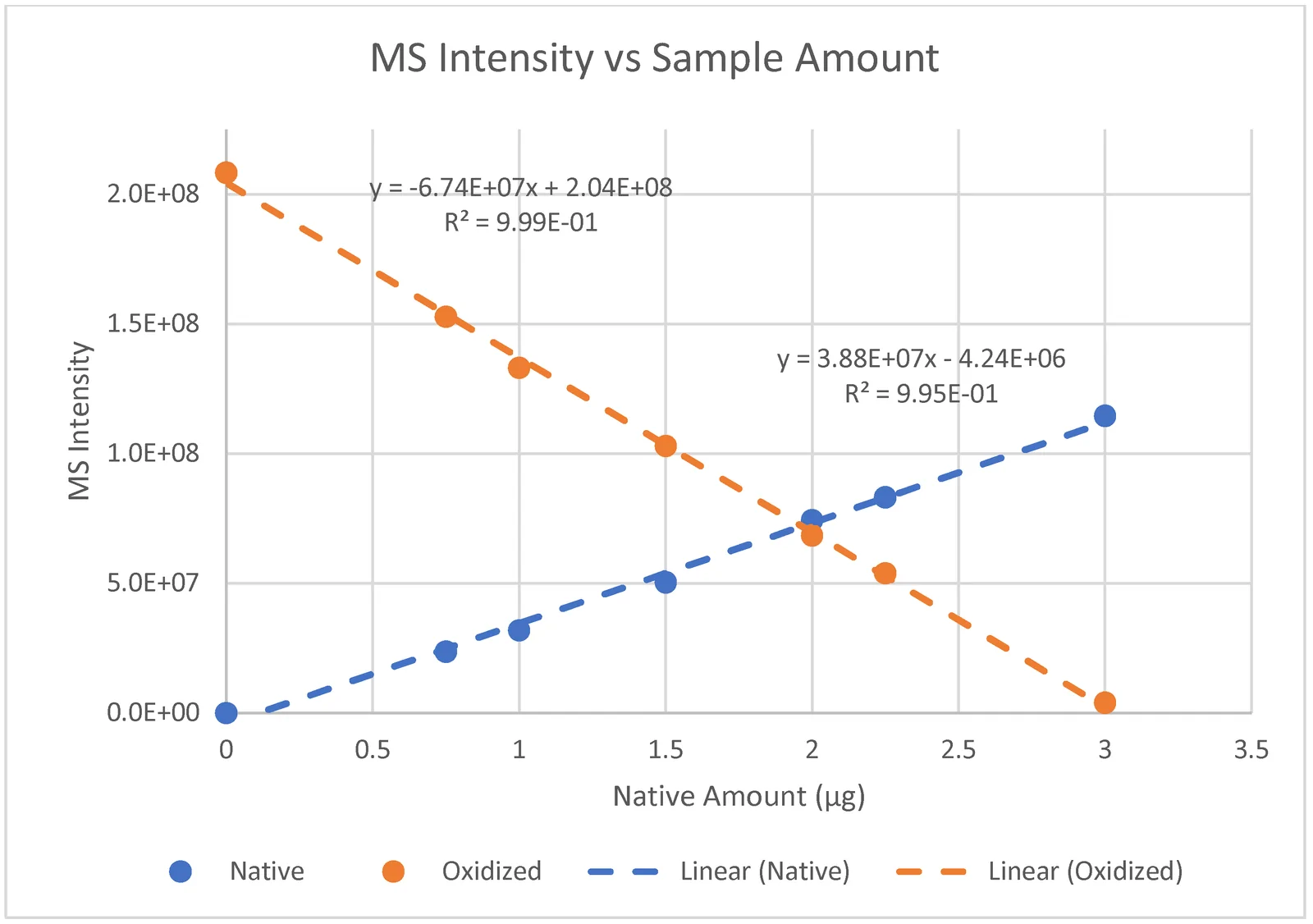
Linearity assessment of native (blue) and oxidized (orange) DTLMISR based on injections of 0–3 µg of adalimumab.

These mixtures corresponded to 3.0, 2.25, 2.0, 1.5, 1.0, 0.75, and 0 µg of oxidized protein, with complementary amounts of the native (non-oxidized) species. As shown in Figure 7, the measured signal responses for both the native and oxidized DTLMISR peptide exhibited excellent linearity across the full analytical range, with coefficients of determination (R^2^) exceeding 0.99. Similar linear behavior was observed for the native and oxidized DIQMTQSPSSLSASVGDR peptide pair (Supplemental Figure S3), with R^2^ values also exceeding 0.98. These results demonstrate proportional response behavior across two representative peptide/PTM pairs and support the quantitative performance of the workflow over the evaluated range of oxidation levels.

Collectively, these results establish a quantitative analytical range of 0–100% for the evaluated oxidation-related attributes in adalimumab. Because this evaluation was performed using representative peptide/PTM pairs, the resulting performance characteristics should not be extrapolated to all monitored PQAs without additional attribute-specific assessment. Nevertheless, the observed linearity across two distinct peptide/PTM pairs demonstrates that the MAM workflow is suitable for reliable quantitative monitoring of representative quality attributes and supports its application for routine attribute-based control.

Error bars are not shown because each condition was analyzed once in a controlled trend study. Method precision and reproducibility were evaluated separately and are presented elsewhere in the manuscript.

#### 2.4.5. Detection Limit (DL) and Quantitation Limit (QL)

DL and QL were determined on an attribute-by-attribute basis, consistent with General Chapter 〈1060〉 guidance, which recognizes that DL and QL in MAMs are inherently attribute dependent. This dependency arises from differences in peptide recovery, chromatographic behavior, and ionization efficiency, even among closely related peptide forms. As a result, sensitivity characteristics established for one attribute cannot be assumed to apply universally across all monitored attributes within a MAM workflow.

Several approaches may be used to establish DL and QL for MS-based assays. One commonly applied strategy follows USP and ICH recommendations, defining DL as a signal-to-noise (S/N) ratio of approximately 3 and QL as an S/N ratio of approximately 10. When applied to high-resolution mass spectrometry data, this approach often yields substantially lower DL and QL values than those achievable with conventional chromatographic or optical detection techniques, due to the high specificity and selectivity of accurate-mass ion detection.

As shown in Figure 6, injection of a 1:1 mixture of adalimumab control digest and oxidation digest produced robust MS responses for both the oxidized and native forms of the DTLMISR peptide. For a total injection corresponding to 1.5 µg of oxidized protein, the oxidized DTLMISR peptide exhibited an S/N of 284,094, while the native DTLMISR peptide exhibited an S/N of 202,404 for an equivalent 1.5 µg protein contribution. Based on these measured S/N values and application of the S/N = 3 and S/N = 10 criteria, the DL was estimated to be 15.8 pg protein for oxidized DTLMISR and 22.2 pg protein for native DTLMISR. Corresponding QL values were estimated to be 52.8 pg protein for oxidized DTLMISR and 74.1 pg protein for native DTLMISR.

The observed difference in sensitivity between the native and oxidized forms of DTLMISR reflects differences in their ionization efficiencies under the applied LC-MS conditions. This finding underscores an important consideration for MS-based MAM measurements: relative quantitation based on peptide signal intensity should not be interpreted as absolute quantitation across different modification states. Instead, quantitative performance such as DL and QL values should be evaluated and controlled at the individual attribute level, with appropriate calibration, consistent with the attribute-centered principles articulated in General Chapter 〈1060〉.

#### 2.4.6. Robustness

General Chapter 〈1060〉 provides experimental guidance for evaluating MAM robustness, including controlled variations in digestion time, incubation temperature, buffer composition, protease source, LC gradient stability, column temperature, and MS source parameters. In this work, these considerations were addressed during method development through method design and prior knowledge, although detailed experimental results are not presented. Overall, the approach yielded consistent attribute quantitation and stable NPD performance, meeting the robustness expectations of both General Chapter 〈1060〉 [42] and ICH Q14 [60] as well as ICH Q2(R2) [58].

In this study, freeze–thaw stability and autosampler hold time were evaluated to assess sample stability under routine QC conditions. These factors, which may arise during sample handling and extended batch analysis, can affect PTM integrity and quantitation performance, and were therefore examined to support method robustness. Supplemental Tables S6 and S7 summarize the robustness results from freeze–thaw stability testing and autosampler stability testing of adalimumab digest. Most PQAs showed good freeze–thaw stability (%RSD < 10%). A few low-abundance peptides—such as the deamidated NSLYLQMNSLR—showed reduced recovery after a single freeze–thaw cycle but exhibited similar recovery across the second and third cycles.

Most PQAs also demonstrated good autosampler stability (%RSD < 10%) even upon reinjection 65 h after the initial injection. However, the oxidized NSLYLQMNSLR peptide showed poor autosampler stability. Prolonged autosampler residence at 5 °C appeared to slightly increase the oxidation level (from 0.42% to 0.56%).

Overall, the robustness results indicate that injecting freshly prepared samples and using relatively short injection sequences are important for obtaining reproducible and reliable MAM results.

#### 2.4.7. New Peak Detection (NPD) Capability

NPD is an important component of the MAM workflow for identifying unexpected or changing attributes, including newly observed signals as well as increases or decreases in existing signals based on defined peak area ratio criteria. In this work, the NPD capability was evaluated using a mixture of 15 heavy-labeled peptides spanning a molecular weight range of approximately 800–2500 Da spiked into the adalimumab digest. The NPD functionality was assessed for its ability to identify peptide-level signals above defined thresholds, including criteria such as minimum intensity or S/N, reproducibility across injections, and consistent elution times. These results indicate that the workflow can flag genuine changes in the peptide map while minimizing false positives from normal analytical variability. The heavy-labeled peptide spike approach was selected as a controlled model system to evaluate NPD functionality and reporting thresholds. However, it does not fully replicate the complexity of product-related degradation products, sequence variants, truncations, glycoform changes, or other unexpected species that may occur in biotherapeutic samples. Using Chromeleon’s Non-Targeted MS processing and optimized parameters (S/N 20, MS noise 80,000, mass accuracy ≤ 6 ppm, charge states 2–5, ratio not between 0–200, MS/MS area ≥ 950,000), Non-Targeted MS detected 18 new peaks (Supplemental Table S8). All corresponded to the 15 spiked unique peptides; the additional peaks were due to alternative charge states or in-source fragments. For example, peaks 17 and 18 represent different charge states of LSSEAPALFQFDLK; peaks 6 and 7 represent different charge states of DIPVPKPK; and peak #5 corresponds to PVPKPK, an in-source fragment of DIPVPKPK. Therefore, the present study demonstrates the ability of the workflow to detect and report unexpected peptide-level signals under controlled conditions. Additional studies using stressed materials and product-related variants would be required to fully establish NPD performance for product-specific QC applications. Although Chromeleon software 7.3.2 was used in this study, the fundamental NPD principles described here are not dependent on a single software platform. Alternative software solutions with comparable feature detection, alignment, filtering, and reporting capabilities may be suitable, although platform-specific parameters may require optimization and qualification.

### 2.5. MAM Analysis of Adalimumab and Etanercept

The qualified in-solution tryptic MAM workflow was applied to two representative biotherapeutics, including the mAb adalimumab and the Fc-fusion protein etanercept, which differ in structure and PTM profiles. These case studies were selected to demonstrate application of the workflow across molecules with differing levels of structural complexity; however, product-specific optimization, qualification and validation may still be required depending on the biotherapeutic and intended application.

#### 2.5.1. Peptide Detectability, Sequence Coverage, and Chromatographic Performance

Consistent peptide detectability and stable chromatographic behavior were observed across replicate LC-MS injections for both molecules. Adalimumab routinely achieved >98% sequence coverage for both light and heavy chains, confirming efficient digestion and low-artifact sample preparation. In contrast, etanercept exhibited 87–93% coverage, with missing regions concentrated in heavily glycosylated segments known to resist tryptic cleavage. Although this level of coverage was sufficient to support the objectives of the present study and monitoring of the selected PQAs, incomplete coverage may limit the ability to detect modifications or unexpected species within regions not represented in the peptide map. Product-specific optimization strategies, including alternative digestion approaches, may be considered when higher coverage is required. These observations are consistent with the more complex fusion protein structure of etanercept. Total ion chromatograms showed high reproducibility for both molecules (Supplemental Figure S4), and the final MAM monitoring panel consisted of 15 peptides for adalimumab and 20 peptides for etanercept.

#### 2.5.2. Deamidation and Succinimide Formation

**Figure 8:**
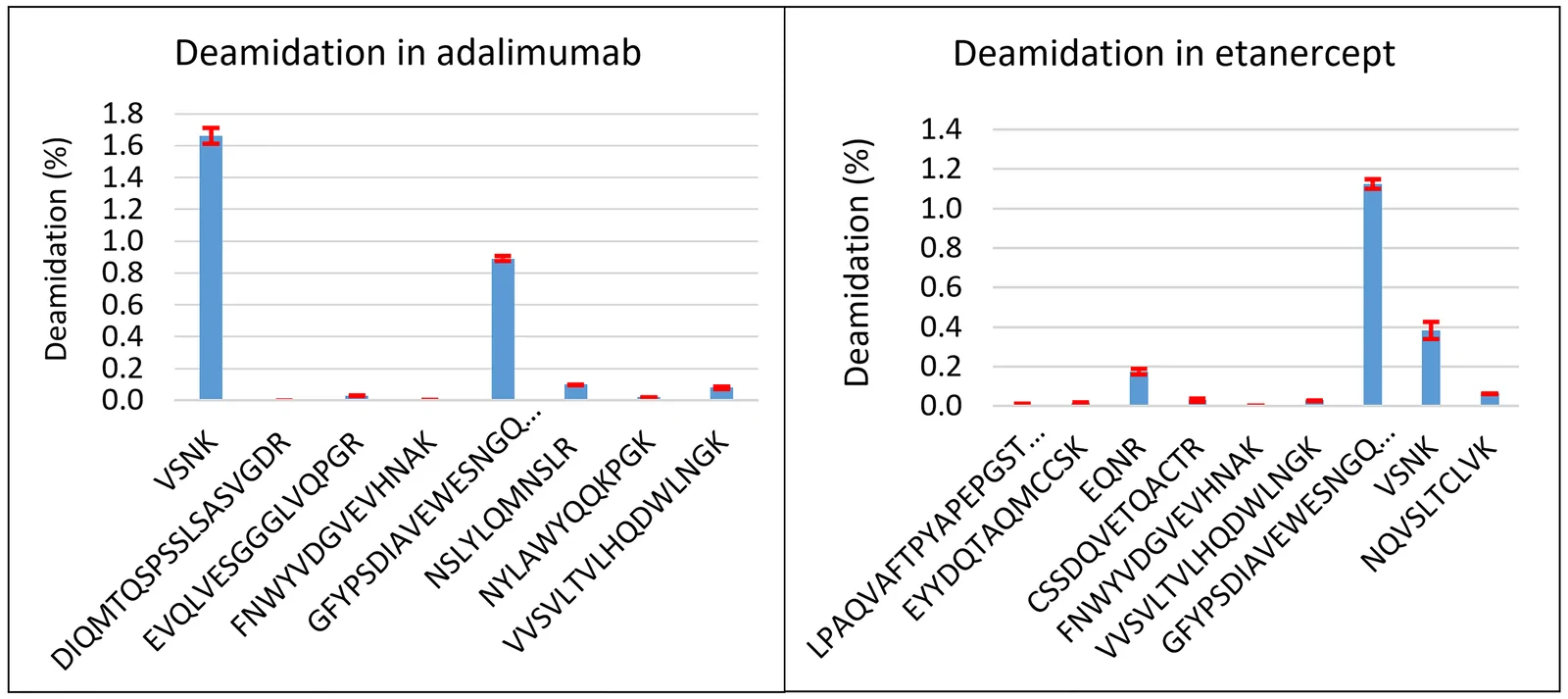
Average relative abundance profiles of deamidation species in adalimumab and etanercept, illustrating comparative levels of these related modifications across the two biotherapeutics. The error bars represent ±1 standard deviation of the MAM measurements from four replicate injections of two sample preparations (2 injections per sample preparation). The sequences for some peptides were abbreviated; see Supplemental Tables S1 and S2 for full sequence.

Deamidation levels were low and reproducible across replicate measurements. In adalimumab, VSNK exhibited the highest degree of deamidation (1.66% ± 0.05%), followed by GFYPSDIAVEWESNGQPENNYK (0.89% ± 0.02%). In etanercept, GFYPSDIAVEWESNGQPENNYK (1.13% ± 0.02%) and VSNK (0.38% ± 0.04%) represented the most modified peptides. All remaining peptides from both molecules exhibited ≤0.10% deamidation (Figure 8).

Succinimide formation followed a similar pattern of reproducibility. In adalimumab, four peptides showed very low levels (≤0.22%), except for GFYPSDIAVEWESNGQPENNYK (1.32% ± 0.02%). For etanercept, CSSDQVETQACTR exhibited the highest succinimide level (4.12% ± 0.36%), whereas the other peptides had levels ≤1.17%. Standard deviations for all peptides were low, indicating stable and repeatable quantitation.

#### 2.5.3. Oxidation of Methionine and Tryptophan Residues

Methionine and tryptophan oxidation were quantified across multiple peptides for each molecule. Adalimumab displayed oxidation levels ranging from 0.001% to 3.84%, with several robustly detected peptides, including DTLMISR and LSCAASGFTFDDYAMHWVR, showing low variability (Supplemental Figure S5). Etanercept exhibited higher oxidation heterogeneity consistent with its structure, with EYYDQTAQMCCSK (3.48% ± 0.48%), VVSVLTVLHQDWLNGK (2.05% ± 0.20%), and DTLMISR (1.49% ± 0.13%) accounting for the majority of observed oxidation. Variability remained low across all monitored peptides (SD = 0.06–0.48%), and no drift was detected across injections.

#### 2.5.4. Terminal Modifications and DP Clipping

C-terminal lysine clipping was the predominant form in both molecules, occurring at 67.58% ± 0.46% in adalimumab and 73.39% ± 0.12% in etanercept (Supplemental Figure S6). Unclipped variants were also consistently detected at expected levels and aligned with orthogonal charge-variant data from conventional assays. For etanercept, a minor amidated C-terminal form (2.43% ± 0.02%) was also detected.

DP clipping was observed at low but reproducible abundances on the same tryptic peptide region for both molecules: 0.31% ± 0.05% for adalimumab (D260) and 0.24% ± 0.04% for etanercept (D290). These consistently low values highlight the workflow’s capability to reliably resolve low-level clipping events (Supplemental Figure S7). For adalimumab, N-terminal pyroglutamate modification was also reproducibly detected at a low level (0.39%).

#### 2.5.5. N-Glycosylation and O-Glycosylation

**Figure 9:**
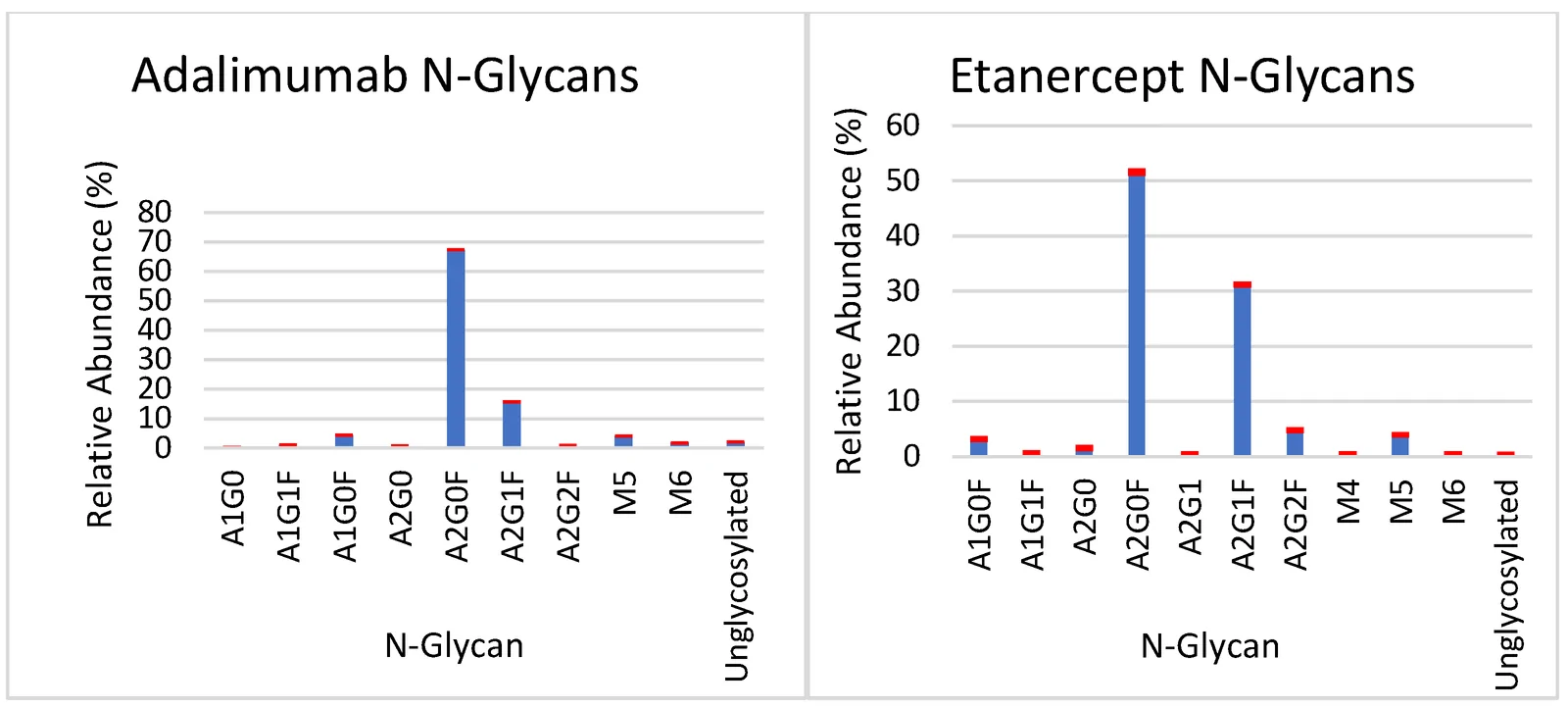
Average relative abundance profiles of selected N-glycans on the glycopeptide EEQYNSTYR in adalimumab and etanercept, as determined by MAM, illustrating comparative glycosylation patterns between the two biotherapeutics. The error bars represent ±1standard deviation of the MAM measurements from four replicate injections of two sample preparations (2 injections per sample preparation).

The shared Fc glycopeptide EEQYNSTYR supported robust profiling of N-glycans for both molecules. Adalimumab and etanercept exhibited 18 and 19 glycoforms, respectively, with A2G0F (G0F) and A2G1F (G1F) as the predominant species in both cases (Figure 9). Relative abundances of most N-glycoforms demonstrated good reproducibility.

In etanercept, N-glycosylation sites in the TNF-α domain (N149, N171) exhibited poor recovery (<1%), preventing confident glycan profiling. This is consistent with known challenges associated with sialylated, ionization-labile peptides [53].

O-glycosylation, unique to etanercept, was reproducibly quantified for multiple sites.

- LPAQVAFTPYAPEPGSTCR: low-level O-glycans at 1.94% (GalNAc-6S-3SG) and 1.82% (GalNAc-3SG), with trace amounts of GalNAc-6S.
- SMAPGAVHLPQPVSTR: four O-glycoforms detected; GalNAc-3SG was predominant (85.82%).
- THTCPAPELLGGPSVFLFPPKPK: three O-glycoforms detected at 0.08–2.26%.

All O-glycans exhibited excellent repeatability (SD ≤ 0.57%; Supplemental Table S9).

#### 2.5.6. Additional Low-Level Modifications

In etanercept, low-level aspartic acid isomerization (<1%) was detected at D300 and D421, and glycation (<1%) was detected at K337 and K346, with K346 exhibiting slightly higher levels. Glycation was quantified using native and miscleaved peptide peak areas, and values were consistent across replicates (Supplemental Figure S8). Although low in abundance, these modifications are reported to influence IgG function and therefore remain relevant for monitoring [50].

### 2.6. Assay Transfer Strategy and Interlaboratory Demonstration

Following completion of method qualification in the sending laboratory, the MAM workflow was transferred to a receiving laboratory to assess interlaboratory reproducibility and evaluate robustness of the locked workflow under varied operating conditions. A structured, risk-based transfer strategy was employed to harmonize procedures, mitigate analytical variability, and ensure consistent execution across sites.

#### 2.6.1. Pre-Transfer Alignment and Method Locking

Preparation for assay transfer focused on harmonization of critical documentation, including SOPs for sample preparation, LC-MS operation, data processing, and result reporting. Locked data-processing methods were distributed to both laboratories, encompassing target peptide lists, retention-time windows, attribute definitions, isotopic-fit criteria, mass-accuracy tolerances, and calculation logic. This ensured analytical equivalence independent of instrument-specific signal characteristics.

A structured pre-transfer assessment was conducted to align critical analytical parameters, predefined acceptance criteria, and system-readiness requirements. Method-transfer acceptance criteria and expected ranges were established prospectively using qualification data generated with USP mAb 001, together with system-readiness data generated in both laboratories. The criteria focused on predefined measures of workflow and attribute performance, including system readiness, peptide detectability, retention-time consistency, and attribute-level comparability, rather than direct matching of raw instrument responses. Early identification of gaps related to instrumentation, software versions, consumables, and laboratory workflows enabled timely corrective actions prior to formal method transfer.

#### 2.6.2. Training and Technical Alignment

Hands-on training was conducted at the receiving laboratory using USP mAb 001 RS as the standardized control material. Analysts from the sending laboratory first demonstrated the complete MAM workflow. Following this, analysts from the receiving laboratory performed the full workflow themselves, including sample preparation, instrument setup, system readiness checks, and data interpretation, under the direct supervision of the analysts from the sending laboratory. To minimize variability, comparable (when possible, identical) reagent lots, standards, and consumables were used across sites. Although the LC-MS systems in the two laboratories were different models, both were from the same vendor and were qualified for comparable performance specifications. These measures helped ensure procedural consistency during transfer to independent operation.

#### 2.6.3. Interlaboratory Evaluation of System Performance

Interlaboratory comparability was evaluated after completion of documentation alignment and training. Key performance metrics included peptide detectability, retention-time precision, quantitation of PTMs (e.g., methionine oxidation and deamidation), and Fc glycan distributions. All data were processed using the same locked, vendor-agnostic workflow.

**Figure 10:**
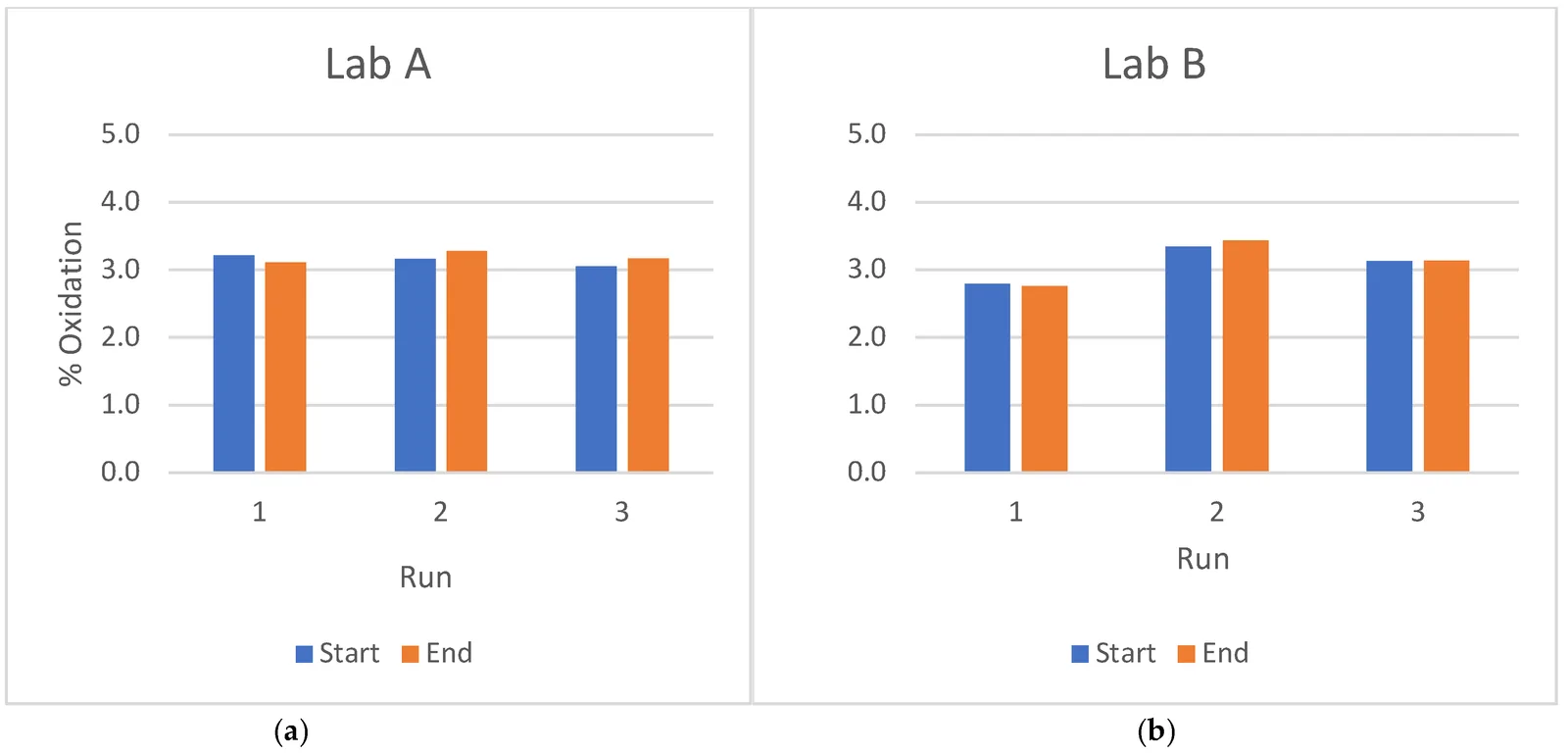
Relative abundance of DTLM(Ox)ISR peptide from USP mAb 001 RS detected by (**a**) Lab A (sending laboratory) and (**b**) Lab B (receiving laboratory). For each run, the blue and orange bars represent the first and last injections, respectively, bracketing the study sample injections. Oxidation levels remained between 2% and 4% at both sites, demonstrating strong interlaboratory reproducibility of the MAM workflow.

System-readiness criteria and sequence-coverage thresholds were met at both sites (see Section 2.3 and Section 2.5.1). Retention-time profiles of core peptides aligned within predefined acceptance windows, and all predefined attribute-level acceptance criteria were met. As an example, the relative abundance of the oxidation-prone DTLMISR peptide from USP mAb 001 RS (reported as DTLM(Ox)ISR) showed close agreement between laboratories, with an average of 3.1% ± 0.1% across analytical sequences without evidence of drift (Figure 10). Similar agreement was observed across instruments, analysts, and multiple operating days, supporting successful transfer of the workflow between laboratories.

#### 2.6.4. Interlaboratory Attribute-Level Comparison

**Figure 11:**
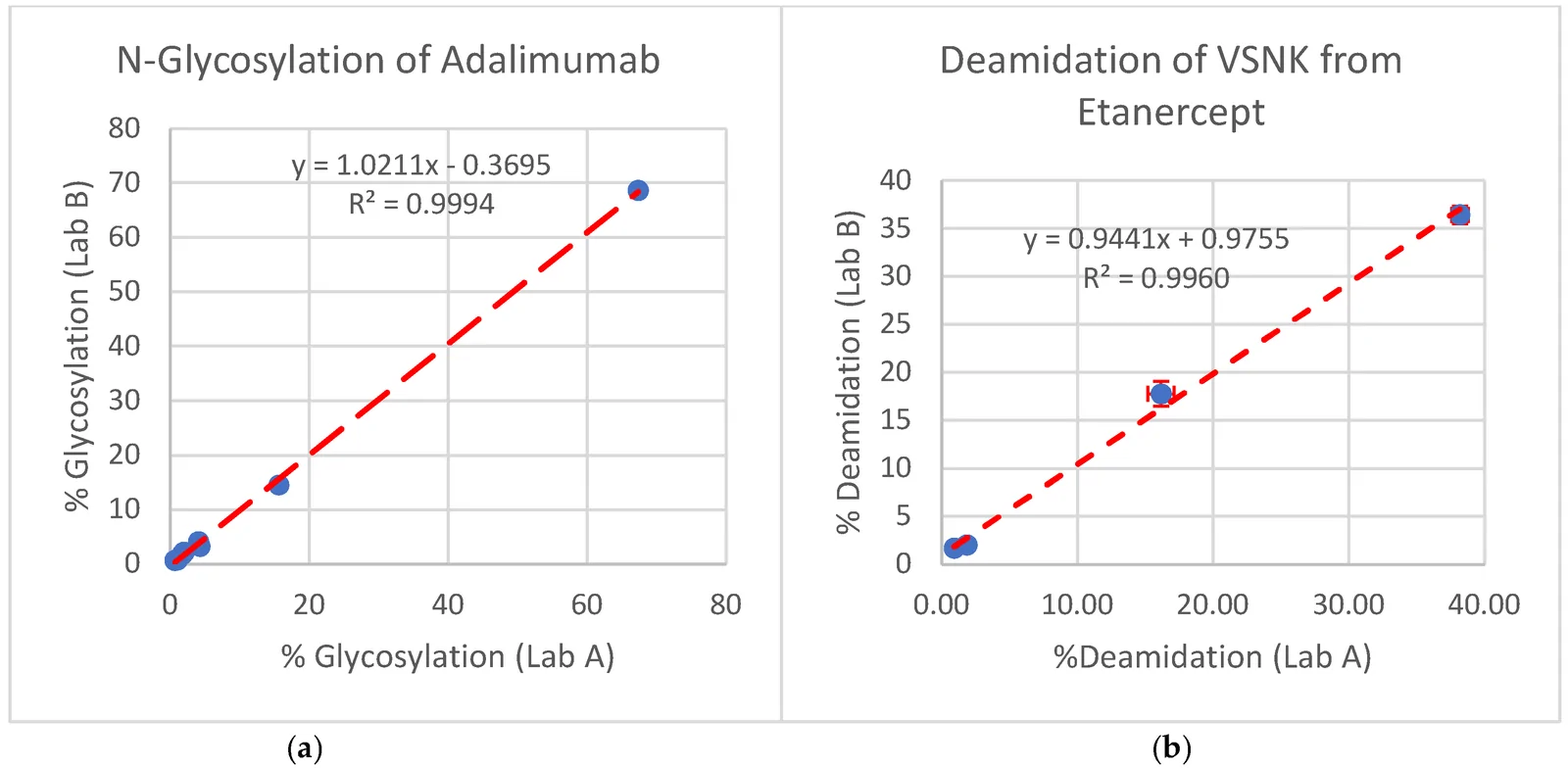
Interlaboratory comparison of (**a**) the relative abundances of N-glycoforms of the EEQYNSTYR peptide in a non-stressed adalimumab sample (error bars are not visible at this scale due to very low standard deviations); and (**b**) the relative deamidation levels of the VSNK peptide in stressed etanercept samples, where error bars represent one standard deviation from four injections of two sample preparations. Blue dots indicate the measured results, and red dashed lines indicate the fitted linear regression trend lines.

Attribute-level comparison further demonstrated excellent agreement between laboratories. Two representative examples are shown in Figure 11. For adalimumab, nine N-301 glycoforms (M5, M6, A1G0F, A1G1F, A2G0, A2G0F, A2G1F, A2G2F, and the unglycosylated form) were detected, and their relative abundance exhibited strong linear correlation between sites (R^2^ = 0.9994) (Figure 11a). Similarly, for etanercept, the deamidation levels of the VSNK peptide measured in thermally and chemically stressed samples were highly comparable between laboratories, yielding an R^2^ value of 0.9960 (Figure 11b). Representative attribute-level acceptance limits used for transfer evaluation are provided in Supplemental Table S10 and were applied prospectively during assessment of transfer success.

Together, these examples demonstrate the ability of the locked MAM workflow to reproducibly quantify diverse quality attributes across laboratories, even for biotherapeutics with distinct molecular architectures and levels of complexity. Other monitored PQAs, including oxidative modifications, N-terminal cyclization, C-terminal Lys variants, and DP clipping, also showed excellent agreement between sites.

#### 2.6.5. Assessment of Transfer Success

Successful transfer was defined by agreement in attribute-level results within predefined acceptance criteria established prior to study execution. No laboratory-specific trends or systematic biases were observed across the evaluated attributes. The strong agreement across sites, despite differences in instrumentation, laboratories, operators, reagent lots, and testing days, demonstrates that the locked MAM workflow can be reliably transferred while maintaining analytical control.

Collectively, these results confirm that harmonized documentation, locked data-analysis methods, targeted technical training, and rigorous system-readiness controls enable successful and reproducible implementation of MAM across laboratories and analytical platforms, supporting its implementation in regulated environments following appropriate product-specific qualification, validation, and lifecycle management activities.

## 3. Discussion

### 3.1. Overview and Context for an MAM Implementation Roadmap

This study describes the development and implementation of a platform-based MAM workflow across two laboratories and demonstrates how a General Chapter 〈1060〉-aligned framework can support the practical implementation of LC-MS-based MAM. While MAM is well recognized for its ability to provide high-resolution monitoring of PQAs, its transition from research-grade characterization workflows to fully governed QC applications has remained challenging. The workflow provides a broadly applicable framework to support performance monitoring, method refinement, and analytical transfer, and serves as a foundation for product-specific optimization, attribute selection, qualification, and validation, which remain essential for adoption of MAM for individual biotherapeutic products in QC environments.

The roadmap presented here is based on practical implementation rather than theoretical considerations and uses two representative protein therapeutics—adalimumab and etanercept—to demonstrate approaches for method development, system readiness, qualification, and interlaboratory transfer.

Although LC-MS platform selection and software configuration remain foundational, this work focuses on practical aspects of MAM implementation that directly influence method performance and control throughout the workflow. Modern high-resolution LC-MS systems provide mass accuracy, resolving power, and chromatographic stability required for peptide-mapping analyses. In this context, instrument capability is considered a qualified baseline, allowing greater emphasis on method control, system suitability, and ongoing performance monitoring.

Similarly, while data-integrity and algorithm stability remain essential in regulated environments, the primary requirement for MAM software is to support reliable automated processing, consistent peak integration, and controlled definition of monitored attributes. This approach emphasizes sustained method performance and operational consistency to support routine use.

### 3.2. Optimization of Sample Preparation

The use of representative molecules in this work highlights practical approaches to minimizing sample-preparation–induced artifacts, a key consideration in MAM workflows [16,28,40]. Peptide mapping, the analytical backbone of MAM, is highly susceptible to artifactual PTMs, particularly methionine oxidation and asparagine deamidation [39]. These artifactual PTMs can obscure true product-related quality differences and therefore must be tightly controlled.

Optimization studies in this project focused on understanding how digestion chemistry, temperature, pH, and reaction time influence artifact formation. Lowering the digestion buffer pH to 7.4 and shortening the reduction/digestion time to under two hours substantially reduced both oxidation and deamidation artifacts while preserving adequate digestion efficiency. In addition, including methionine as an oxidation scavenger further suppressed artifactual methionine oxidation without compromising digestion [22].

The same general sample-preparation approach was also evaluated in other mAbs, including rituximab and bevacizumab, with comparable trends observed in artifact control. Collectively, these results provide a practical framework for minimizing sample preparation–induced artifacts and enabling more accurate assessment of PQAs in MAM workflows.

### 3.3. System Readiness as an Integrated Performance Check

One of the most impactful components of the General Chapter 〈1060〉 framework is the concept of system readiness testing. While instrument-level checks ensure baseline hardware function, system readiness evaluates the *entire* analytical process—sample preparation, chromatography, mass spectrometry, and data processing—prior to generating reportable QC results. In this study, the USP mAb 001 RS served as a consistent benchmark for assessing digestion reproducibility, retention-time stability, signal robustness, and sensitivity to labile PTMs. This material provided a molecule-independent means of verifying that the MAM workflow was functioning reliably as intended at the time of use. Importantly, predefined acceptance criteria established prior to study execution and longitudinal trending enabled early detection of subtle performance drifts. For example, oxidation levels in sentinel peptides such as DTLMISR—highly sensitive to oxygen exposure and instrument cleanliness—served as practical indicators of system readiness. This approach emphasizes that MAM fitness must be demonstrated through holistic performance evaluation rather than relying solely on hardware qualification. The system readiness strategy presented here thus serves as a model for laboratories seeking to operationalize these recommendations.

In this context, system readiness and system suitability serve different but interconnected roles in enabling broader adoption of the MAM workflow. System readiness establishes confidence that the integrated analytical platform is operating consistently and producing interpretable results, supporting its application in characterization and other exploratory settings. Data generated through system readiness evaluations can subsequently inform the definition of method-specific system suitability criteria, focusing on the parameters most critical to routine testing. With these suitability criteria in place and supported by full method validation, the assay can be transitioned for use in QC environments, including release and stability testing.

### 3.4. Attribute-Centered Method Qualification

General Chapter 〈1060〉 emphasizes that qualification of MAM should be attribute-focused and should consider analytical performance across the full peptide-mapping workflow, rather than relying solely on global method-level metrics. This attribute-centered perspective guided the qualification strategy applied to the adalimumab and etanercept MAM assays described in this work. Performance was assessed at the individual PQA level, recognizing that different attributes may exhibit distinct chromatographic behavior, ionization efficiency, or susceptibility to matrix effects. For certain qualification parameters, including accuracy, linearity, DL, and QL, representative peptides were used as model analytes to demonstrate the qualification strategy. While these results provide evidence of workflow capability, other peptides and PTMs may exhibit different analytical behavior and may require additional attribute-specific evaluation.

In this study, method qualification was performed using traditional qualification and validation characteristics, including specificity, accuracy, precision, linearity, range, and QL/DL, evaluated in accordance with ICH principles and relevant USP guidance. These characteristics were applied on an attribute basis rather than as a single aggregate assessment. Controlled sample mixtures, forced-degradation materials, and reference standards were used, as appropriate, to assess quantitative performance under conditions relevant to the intended analytical use. While the present study establishes the analytical framework required for MAM implementation and qualification, comprehensive evaluation of the full unstressed and stressed sample set, together with comparisons to conventional orthogonal methods, will be important for demonstrating the ability of the workflow to detect and quantify product changes in product-specific applications. The specificity studies performed in this work were intended to support platform-level qualification and demonstrate discrimination against formulation components and unrelated protein matrices. Additional evaluation of product-related variants, degradation products, process- and product-related impurities, stressed samples, and other molecule-specific interferences would be required during product-specific validation prior to routine QC implementation. For low-abundance attributes or those prone to interference, particular attention was given to precision and accuracy near the QL/DL to ensure confidence in quantitative interpretation.

NPD was incorporated into the qualification framework as the impurity-detection component of the MAM workflow, consistent with General Chapter 〈1060〉 principles. Thresholds for signal intensity, fold change, isotopic pattern matching, and charge state consistency were defined to balance sensitivity to true structural changes with control of false positives. Spiking a range of known, unrelated peptides into peptide digests represents a practical and well-controlled strategy for evaluating NPD performance, enabling assessment of detection sensitivity, reporting thresholds, and method robustness. However, this controlled model does not fully reproduce the complexity of product-related degradation products, sequence variants, truncations, glycoform changes, or other species that may arise in biotherapeutic products. Therefore, the NPD results presented here should be viewed as demonstrating workflow functionality rather than providing a comprehensive assessment of NPD performance for all potential product-related impurities or degradation products.

More broadly, this article focuses on presenting assay qualification data, which establish analytical feasibility, attribute-level performance, and method understanding while providing a foundation for subsequent product-specific studies involving stressed samples and orthogonal analytical comparisons. These qualification results are intended to inform subsequent definition of system suitability acceptance criteria and to support progression toward full validation as part of the broader MAM lifecycle and QC implementation roadmap.

### 3.5. MAM Performance for Adalimumab and Etanercept

The performance of the MAM workflow for both adalimumab and etanercept was consistent with previously reported studies supporting its application for product characterization and analytical comparability assessment [61]. Evaluation of representative peptides demonstrated reliable detectability, retention time consistency, and repeatability across replicate digestions and injections, supporting robust performance for targeted PQA monitoring.

For adalimumab, PTM profiles were evaluated for key attributes, including N-glycosylation, oxidation, deamidation/succinimide formation, N-terminal pyroglutamate formation, and C-terminal lysine clipping. The relative levels obtained in this study were generally consistent with reported values [61], as illustrated by comparable levels of A2G0F (67.3% in this study vs. ~67% in literature), C-terminal lysine clipping (~67.6% vs. ~67%), and NSLYLQMNSLR deamidation (0.3% vs. ~0.2%). For certain PQAs, such as methionine oxidation of the DTLMISR peptide, slightly higher levels were observed in this study (2.8% vs. ~1.8%), likely attributable to differences in material source and storage conditions rather than method variability.

For etanercept, a similar set of PQAs was evaluated, along with the addition of etanercept-specific attributes such as sialylation and O-glycosylation. Comparable results were obtained for most PQAs [62], with close agreement for representative peptides such as oxidation of EEMTK (0.3% vs. ~0.3%) and WQQGNVFSCSVMHEALHNHYTQK (0.3% vs. ~0.2%), as well as deamidation of the PENNY peptide (~1.2% vs. ~1.0%). O-glycosylation of the LPAQVAFTPYAPEPGSTCR peptide was also comparable (GalNAc-3SG: 1.8% vs. ~2%; GalNAc-6S-3SG: 1.9% vs. ~2%). However, sialylated glycopeptides were not detected in this study, in contrast to reported levels (e.g., ~55% A2S1G1 on the TNFR peptide). This difference is likely attributable to sample-preparation conditions, particularly the shorter digestion time used in this study (1 h versus ~4 h in the reference method), which may limit the generation or detectability of sialylated species.

Overall, these findings support the use of the standardized MAM workflow for robust characterization of selected PQAs. At the same time, they indicate that certain attributes—particularly sialylated glycopeptides in etanercept—may require molecule-specific optimization of sample preparation and analytical conditions. The results further provide a basis for continued method refinement, including the establishment of system suitability criteria and evaluation for use in more regulated environments.

### 3.6. Method Transfer Between Laboratories and LC-MS Platforms

This study facilitated identification of workflow components that were inherently robust, such as the optimized sample-preparation steps, as well as elements that required further optimization to achieve comparable results across sites, including LC-MS ion-source parameters. Because the systems used in this study shared similar atmospheric-pressure ionization designs, ionization behavior was largely comparable. However, modest differences in baseline sensitivity required optimization of ion-source temperature and ion-transfer settings, particularly to avoid artifactually inflating or suppressing glycopeptide-related PTMs. These adjustments are fully consistent with General Chapter 〈1060〉’s recognition that LC-MS conditions may require refinement during method transfer to ensure fitness-for-purpose. Harmonization of attribute definitions, processing logic, and readiness criteria ensured that results generated across laboratories were analytically comparable. Transfer acceptance criteria were established prospectively using qualification data, expected analytical variability, and the intended purpose of the transfer assessment, enabling objective evaluation of transfer success without post-study adjustment of thresholds. The strong interlaboratory correlation observed for glycan relative abundance and deamidation trends further supports that the MAM workflow can be successfully transferred between systems with differing sensitivity characteristics while maintaining appropriate analytical control.

Several practical and technical insights were identified during assay transfer, emphasizing the importance of procedural harmonization, alignment of analytical expectations, and proactive mitigation of software- or instrument-specific variables.

- Documentation and protocol control: Standardize and fully document SOPs for sample preparation, LC-MS acquisition, and data processing, with appropriate version control maintained across sites.
- Critical method steps: Clearly define steps affecting analytical outcomes, including digestion conditions, sample handling, MS source tuning, optional UV use, and peak-integration rules.
- Reporting and acceptance criteria: Use harmonized reporting templates and predefined thresholds for sequence coverage, retention-time windows, and system readiness metrics.
- Instrument variability management: Establish common, predefined performance and attribute-level acceptance criteria prospectively using qualification data and expected analytical variability. Instrument-specific tuning and system-readiness verification should be used to confirm that each platform can meet these common criteria without changing the criteria themselves.
- Sample integrity: Verify sample condition upon receipt and confirm that storage and shipping conditions have not compromised sample integrity.
- Technical background and training: Provide cross-site training on molecule attributes, PQAs, and the MAM workflow to ensure consistent scientific understanding.
- Instrument setup and alignment: Conduct tuning, calibration, and verification runs, followed by alignment on optional parameters such as MWCO filter type and methionine quenching strategy.
- System readiness: Establish a common reference standard (RS) and predefined acceptance criteria to assess digestion performance, chromatographic stability, MS sensitivity, and critical PTM levels, ensuring consistent system suitability across laboratories.
- Software considerations: Although the principles of the workflow are broadly software-independent, attention should be given to software-specific differences in feature detection, peak integration, alignment algorithms, isotopic-fit calculations, reporting thresholds, and data management functionality. Software-specific parameters may require optimization and qualification to ensure comparable performance. Continued development of vendor-neutral software workflows and standardized data-review practices may further facilitate broader adoption and transferability of MAM across laboratories and platforms.

### 3.7. Broader Implications and Future Directions

The roadmap presented here provides both a practical template and strategic guidance for broader adoption and implementation of MAM across biopharmaceutical organizations. Several future developments could further improve scalability and robustness. Automated or semi-automated sample-preparation systems may reduce operator-dependent variability, one of the most common sources of workflow inconsistency. Expansion of publicly available reference materials beyond USP mAb 001 RS to include molecules with diverse PTM liabilities and structural formats (e.g., mAbs and fusion proteins) would support more versatile system-readiness benchmarking. Enhanced informatics controls, including version locking, automated audit-trail review, secure method-configuration management, and computation-level reproducibility tools—such as method hashing, where a unique digital fingerprint is generated from all key method parameters and processing settings—can help ensure that identical data-processing workflows are applied consistently across analyses and sites. As mass-spectrometry platforms evolve, improvements in robustness, cycle time, tolerance to varied matrices, and the increasing availability of validated analysis software are expected to further reduce operational barriers for adoption in QC environments.

### 3.8. Integrative Perspective

Overall, this study demonstrates that MAM can be effectively developed, optimized, qualified, transferred, and sustained when implemented under a lifecycle framework aligned with General Chapter 〈1060〉. The primary contribution of this work is the systematic integration and demonstration of established MAM concepts within a practical implementation framework for regulated laboratory environments. Through real-world case studies of adalimumab and etanercept, this work illustrates how laboratories can establish scientifically sound and operationally robust MAM workflows that are suitable for characterization, system readiness assessment, method qualification, and analytical transfer, while providing a foundation for subsequent product-specific validation and future QC implementation. The roadmap emphasizes workflow elements that most directly influence analytical performance and robustness throughout the MAM lifecycle, while recognizing that other components, though still essential (e.g., instrument qualification), are not the primary differentiators of MAM robustness. While full method validation remains a critical requirement for routine QC use, the qualification approach described here provides the foundation to inform system suitability development and support progression toward full validation as part of an integrated MAM implementation strategy for regulated manufacturing.

## 4. Materials and Methods

### 4.1. Materials

USP mAb 001 RS (Cat. No. 1445539) was obtained from the U.S. Pharmacopeia (Rockville, MD, USA). Adalimumab and etanercept were obtained from their respective manufacturers, AbbVie Inc. (North Chicago, IL, USA) and Intas Pharmaceuticals Ltd. (Ahmedabad, Gujarat, India). Tris (hydroxymethyl)aminomethane (Tris), ethylenediaminetetraacetic acid (EDTA), guanidine hydrochloride, dithiothreitol (DTT), iodoacetamide (IAM), L-methionine, sodium hydroxide solution (50% *w*/*w*), Optima LC/MS-grade water, Optima LC/MS-grade water containing 0.1% formic acid, and Optima LC/MS-grade acetonitrile containing 0.1% formic acid were purchased from Thermo Fisher Scientific (Waltham, MA, USA).

Trypsin (sequencing grade, MS Gold, and MS Platinum) was obtained from Promega Corporation (Madison, WI, USA). Amicon Ultra 0.5 mL centrifugal filters (10 kDa MWCO) were sourced from MilliporeSigma (Burlington, MA, USA). Milli-Q water produced using an Advantage A10 system (MilliporeSigma) was used for preparation of all reagent solutions except LC mobile phases.

Chromatographic analyses were performed using an Accucore C18 peptide column (2.1 × 150 mm, 1.5 µm) coupled to a Vanquish UHPLC system and an Orbitrap Exploris 480 (or Exploris 240) mass spectrometer (Thermo Fisher Scientific).

### 4.2. Sample Preparation and Enzymatic Digestion

The following procedure describes the optimized method used for all sample analyses; pre-optimization conditions evaluated during method development are also described. During development, multiple sample-preparation and instrument parameters were assessed to improve robustness and minimize artifactual modifications based on established peptide-mapping and MAM principles. Approximately 70–100 µg of each therapeutic protein was mixed with 95 µL of denaturation buffer (pH 7.4) containing 8 M guanidine hydrochloride, 10 mM Tris, 0.1 mM EDTA, and 10 mM L-methionine. Reduction was initiated by adding 2 µL of 500 mM DTT, followed by incubation at 37 °C for 30 min. After cooling to room temperature, alkylation was performed by adding 4 µL of 500 mM IAM and incubating the sample in the dark for 20 min.

Reduced and alkylated samples were buffer-exchanged three times into 100 mM Tris (pH 7.4) containing 10 mM L-methionine using Amicon 10 kDa MWCO centrifugal filters operated at 14,000 rcf. The final retentate volume was adjusted to approximately 140–170 µL. Trypsin (7–10 µg, prepared at 1 mg/mL) was added at an enzyme-to-protein ratio of 1:10, and samples were digested at 37 °C for 1 h. Enzymatic digestion was quenched by addition of formic acid to a final concentration of 0.1%, yielding a final digest concentration of approximately 0.5 mg/mL. Digests were analyzed immediately to avoid freeze–thaw-induced modifications.

During method optimization, alternative conditions were evaluated, including denaturation at room temperature, use of denaturation and digestion buffers with and without 10 mM L-methionine, different grades of sequencing-grade trypsin, digestion times ranging from 0.5 to 18 h, and varying denaturation temperatures. The conditions described above were selected based on overall analytical performance and robustness and were applied consistently for all reported analyses.

### 4.3. LC-MS Analysis

For LC-MS analysis, 3–5 µg of each digest was injected onto a Thermo Scientific Vanquish Horizon UHPLC system (Thermo Fisher Scientific) equipped with an Accucore Vanquish C18+ column (2.1 × 150 mm, 1.5 µm) maintained at 50 °C. Peptide separations were performed at a flow rate of 0.25 mL/min over a 115 min gradient. Mobile phase A consisted of 0.1% formic acid in water, and mobile phase B consisted of 0.1% formic acid in acetonitrile. The stepped gradient was as follows: 99% A (0–5 min), 90% A (6 min), 65% A (70 min), and 10% A (72–77 min), followed by re-equilibration at 99% A (79–81 min), intermediate ramps to 90% and 55% A (83.5–91.5 min), a second high-organic step at 10% A (93–99 min), and final re-equilibration to 99% A (101–115 min). Unless otherwise stated, experiments were performed using replicate sample preparations and replicate injections. For studies intended to assess analytical trends rather than method precision, a single sample preparation and a single injection were used.

Mass spectrometric detection was performed using an Orbitrap Exploris 480 or Exploris 240 mass spectrometer (Thermo Fisher Scientific). The vaporizer and ion-transfer tube temperatures were maintained at 225 °C, with a spray voltage of +3.4 kV and Easy-IC internal calibration enabled. Full MS spectra were acquired over the *m*/*z* range of 200–1800 at a resolution of 120,000. Data-dependent MS/MS scans were acquired at a resolution of 15,000 using stepped normalized collision energies of 35% and 40% and were used for PTM identification and PQA selection. Routine MAM analyses for attribute monitoring were performed using MS-only data acquisition. During method optimization, the ion-source temperature was evaluated over a range of 220–350 °C.

For interlaboratory comparability evaluations, thermally stressed samples were prepared by incubation at 40 °C for up to six weeks, while chemically stressed samples were generated by incubation at high pH (11.0) for up to seven days. Data acquisition at the second laboratory was performed using an Orbitrap Exploris 240 mass spectrometer. Interlaboratory assessment criteria, including system readiness requirements and attribute-level acceptance limits, were defined prior to execution of the transfer study based on qualification data and expected analytical variability.

Data processing for PQA selection was performed using Biopharma Finder 5.3 software (Thermo Fisher Scientific), while targeted PQA monitoring and NPD evaluation were performed using Chromeleon 7.3.2 software (Thermo Fisher Scientific) with predefined processing parameters locked prior to qualification and transfer studies. Although Biopharma Finder and Chromeleon were used in this study, the workflow is not intended to be restricted to a specific software platform. Alternative software solutions providing equivalent functionality for feature detection, PQA monitoring, NPD analysis, and reporting may also be suitable following appropriate evaluation, qualification, and validation, as applicable.

Unless otherwise specified in the figure captions, figures and graphical data presentations were prepared using Microsoft^®^ Excel^®^ for Microsoft 365 MSO (Version 2608, Microsoft Corporation, Redmond, WA, USA).

## 5. Conclusions

This study presents a practical roadmap for implementing LC-MS-based MAM, using adalimumab and etanercept as representative molecules to demonstrate applicability for mAbs and Fc-fusion proteins. The proposed roadmap spans method development, system readiness, attribute-centered method qualification, and interlaboratory transfer, and outlines a pathway toward broader implementation of MAM for characterization and future QC applications. The workflow provides a broadly applicable implementation framework that can support performance monitoring, analytical transfer, and subsequent product-specific optimization, PQA selection, acceptance criteria definition, qualification, validation, and lifecycle management for individual biotherapeutics.

Method development studies demonstrated that carefully controlled sample-preparation conditions are essential for generating reliable, artifact-controlled PQA measurements. Systematic adjustments of digestion pH, incubation duration, buffer composition, and incorporation of methionine as an oxidation scavenger significantly reduced preparation-induced PTMs (such as methionine oxidation and asparagine deamidation), while preserving digestion efficiency. Optimization of LC-MS acquisition parameters, including ion source and ion transfer settings, ensured stable retention time, peptide coverage, and glycopeptide quantitation performance across both test molecules. Although the standardized workflow performed well for both molecules, certain attributes, particularly those associated with heavily glycosylated regions, O-glycosylation, or sialylated species, may require additional molecule-specific optimization to achieve the desired analytical coverage and performance.

A robust system readiness strategy, anchored by a publicly available mAb reference standard (USP mAb 001 RS), enabled an end-to-end evaluation of workflow performance at the time of analysis, confirming digestion consistency, chromatographic stability, mass accuracy, and data-processing functionality. Beyond confirming that the MAM platform was fit for purpose during qualification studies and routine analytical use, the system readiness results provided a quantitative and empirical basis for defining method-specific system readiness parameters and acceptance criteria. This framework provides a foundation for future method validation and outlines elements that would be required to support deployment of the assay in cGMP QC applications, including appropriate system suitability criteria for release and stability testing, consistent with the lifecycle principles described in General Chapter 〈1060〉.

This study presents the development and qualification of a broadly applicable MAM workflow using representative mAb and Fc-fusion protein case studies that is aligned with ICH principles and the attribute-centric framework of General Chapter 〈1060〉 and can serve as a practical starting point for broader implementation. Attribute-level evaluations demonstrated consistent specificity, precision, quantitative performance, and sensitivity for targeted PQA monitoring. In addition, NPD assessments supported the method’s ability to detect emerging or changing species under controlled conditions using representative peptide spike experiments. Collectively, these results establish a robust foundation for MAM qualification and support the use of attribute-centric approaches for biopharmaceutical analytical characterization and quality assessment.

Finally, assay transfer studies showed that a well-controlled MAM workflow can be successfully transferred between laboratories and LC-MS systems when supported by harmonized attribute definitions, standardized data-processing logic, predefined acceptance criteria, a common reference standard, system readiness criteria, and appropriately tuned instrument parameters. Consistent attribute-level results across sites and instruments support the robustness and transferability of the workflow.

Overall, this work demonstrates that LC-MS-based MAM can be effectively developed, qualified, transferred, maintained, and operated within a structured implementation framework. The results demonstrate a practical attribute-centric approach to MAM implementation and establish a strong technical and operational foundation for broader implementation in regulated laboratory environments. The qualification and system readiness results presented here establish a solid technical and operational foundation for subsequent product-specific studies involving stressed samples, orthogonal analytical comparisons, system suitability criteria development, and full validation as methods progress toward routine QC implementation. Looking ahead, broader adoption will benefit from continued advances in automated sample preparation, expanded reference materials for benchmarking, enhanced data-integrity controls, and greater harmonization across instrument platforms. Continued development of vendor-neutral software capabilities and standardized data-review practices will facilitate consistent implementation across laboratories and software platforms. Together, these developments will further strengthen robustness, scalability, and regulatory confidence, supporting the integration of MAM into modern quality strategies for complex biotherapeutic products.

## Figures and Tables

**Table 1:** Representative attributes monitored for MAM analysis of adalimumab and etanercept.

Attribute	Peptide/Residue	Typical Site	Relevance
Methionine oxidation	Met residues (e.g., Fc Met252, Met428; variable region Mets)	Fc, CDR, hinge	Impacts structural stability, Fc receptor binding, and potency
Deamidation	Asn residues (e.g., deamidation-prone motifs such as NG, NS)	CDR and Fc	Charge heterogeneity
DP clipping	Asp-Pro (DP) motifs	Hinge/Fc or sequence-specific site	Leads to backbone cleavage, affecting integrity and stability
N-glycosylation	Asn297 (Fc conserved site)	Fc region	Critical for effector function (ADCC/CDC), stability, and clearance
O-glycosylation	Ser/Thr residues (etanercept-specific sites)	TNFR domain (etanercept)	Influences pharmacokinetics and molecular heterogeneity
Sequence coverage	Full peptide map	Entire sequence	Identity confirmation

**Table 2:** Key sample-preparation parameters evaluated during method development.

Parameter	Conditions Tested	Rationale
Denaturation and reduction conditions	Guanidine HCl (8.0 M), Tris-HCl (100 mM, pH 7.4), 0.1 mM EDTA, 5 mM DTT, with and without 10 mM methionine, incubation time: 30 min, RT, 37 °C	Ensure unfolding without artifacts
Alkylation	15 mM iodoacetamide, incubation time: 30 min RT in the dark	Prevent disulfide scrambling
Digestion time	Limited range (e.g., 0.5–18 h)	Balance completeness and artifactual PTMs
Trypsin activity	Sequencing grade, gold MS-grade, platinum MS-grade, etc.	Control digestion reproducibility

## Data Availability

The original contributions presented in this study are included in the article/Supplementary Materials. Further inquiries can be directed to the corresponding author.

## Supplementary Materials

The following supporting information can be downloaded at https://www.mdpi.com/article/10.3390/ph19101648/s1. Figure S1: Relative abundance of selected adalimumab quality attributes under six sample preparation conditions; Figure S2: Chromatogram showing the elution profile of system readiness peptides, with retention time evenly distributed across the chromatographic gradient; Figure S3: Linearity assessment of native (blue) and oxidized (orange) DIQMTQSPSSLSASVGDR based on injections containing 0–3 µg adalimumab; Figure S4: Total ion chromatograms (TICs) from replicate analyses of adalimumab (top panels (a) and (b)) and etanercept (bottom panels (c) and (d)); Figure S5: Peptide-level oxidation susceptibility profiles of adalimumab (a) and etanercept (b), highlighting differences in oxidation-prone regions across the two therapeutic proteins; Figure S6: Relative abundance profiles of C-terminal modifications in adalimumab and etanercept measured by MAM; Figure S7: Relative abundance profiles of DP clipping in adalimumab and etanercept quantified by MAM, demonstrating consistent measurement of DP clipping across both therapeutic proteins; Figure S8: Mean relative percent modification for each monitored peptide in etanercept. Table S1: Selected Adalimumab Quality Attributes Identified by LC-MS/MS Peptide Mapping; Table S2: Selected Etanercept Quality Attributes Identified by LC-MS/MS Peptide Mapping; Table S3: Example System Readiness Summary Table Showing Evaluated Parameters; Table S4: Precision Results for Adalimumab Product Quality Attributes (PQAs); Table S5: Precision Results for Etanercept Product Quality Attributes (PQAs); Table S6: Robustness Assessment of Freeze-Thaw Stability of Adalimumab Digest Samples; Table S7: Robustness Results from Autosampler Stability Test of Adalimumab Digest Samples with Reinjection after 65 hours; Table S8: New Peak Species Detected in PRTC-Spiked Humira Digest Samples; Table S9: Summary of Average O-glycosylation Relative Abundances (%PTM) for Different Glycopeptides in Etanercept; Table S10: Representative MAM System Readiness Criteria and Attribute-Level Transfer Acceptance Limits Used During Interlaboratory Assessment.

## Abbreviations

The following abbreviations are used in this manuscript:

ADCC	Antibody-Dependent Cellular Cytotoxicity
CDC	Complement-Dependent Cytotoxicity
CDR	Complementarity determining region
CQA	Critical quality attribute
DL	Detection Limit
DP	Drug Product
DTT	Dithiothreitol
DS	Drug Substance
EDTA	Ethylenediaminetetraacetic Acid
FDA	U.S. Food and Drug Administration
Fc	Fragment Crystallizable Region
Fuc	Fucose
Gal	Galactose
GlcNAc	N-acetylglucosamine
GMP	Good Manufacturing Practice
HRAM	High-Resolution Accurate-Mass
HRMS	High-Resolution Mass Spectrometry
IAM	Iodoacetamide
ICH	International Council for Harmonisation
IgG	Immunoglobulin G
LC-MS	Liquid Chromatography-Mass Spectrometry
mAb	Monoclonal Antibody
MAM	Multi-Attribute Method
Man	Mannose
MS	Mass Spectrometry
MS/MS	Tandem Mass Spectrometry
MWCO	Molecular Weight Cutoff
NPD	New Peak Detection
PQA	Product Quality Attribute
PTM	Post-Translational Modification
QC	Quality Control
QL	Quantitation Limit
Q2(R2)	ICH Q2(R2) Validation of Analytical Procedures
Q14	ICH Q14 Analytical Procedure Development
RS	Reference Standard
RT	Room Temperature
RT	Retention Time
RSD	Relative Standard Deviation
SNFG	Symbol Nomenclature for Glycans (SNFG)
SOP	Standard Operating Procedure
TNF	Tumor Necrosis Factor
TNFR	Tumor Necrosis Factor Receptor
Tris	Tris(hydroxymethyl)aminomethane
UHPLC	Ultra High-Performance Liquid Chromatography
USP	United States Pharmacopeia

## References

[1] Xu Y., Wang D., Mason B., Rossomando T., Li N., Liu D., Cheung J.K., Xu W., Raghava S., Katiyar A. (2019). Structure, heterogeneity and developability assessment of therapeutic antibodies. mAbs.

[2] Kim W., Kang K.H., Suh J.-H., Yeh M.-K., Chen Y.-C. (2018). Characterization of Biopharmaceuticals Focusing on Antibody Therapeutics. Biopharmaceuticals.

[3] Beck A., Wagner-Rousset E., Ayoub D., Van Dorsselaer A., Sanglier-Cianferani S. (2013). Characterization of therapeutic antibodies and related products. Anal. Chem..

[4] Kozlowski S., Swann P. (2006). Current and future issues in the manufacturing and development of monoclonal antibodies. Adv. Drug Deliv. Rev..

[5] Saitoh S. (2018). The Identification of Critical Quality Attributes (CQAs) for the Development of Antibody Drugs. Yakugaku Zasshi.

[6] Liu H., Ponniah G., Zhang H.M., Nowak C., Neill A., Gonzalez-Lopez N., Patel R., Cheng G., Kita A.Z., Andrien B. (2014). In vitro and in vivo modifications of recombinant and human IgG antibodies. mAbs.

[7] Torosantucci R., Schoneich C., Jiskoot W. (2014). Oxidation of therapeutic proteins and peptides: Structural and biological consequences. Pharm. Res..

[8] Houel S., Hilliard M., Yu Y.Q., McLoughlin N., Martin S.M., Rudd P.M., Williams J.P., Chen W. (2014). N- and O-glycosylation analysis of etanercept using liquid chromatography and quadrupole time-of-flight mass spectrometry equipped with electron-transfer dissociation functionality. Anal. Chem..

[9] Martinez V.P.M., Tierrablanca-Sanchez L., Espinosa-de la Garza C.E., Juarez-Bayardo L.C., Pina-Lara N., Santoyo G.G., Perez N.O. (2020). Functional analysis of glycosylation in Etanercept: Effects over potency and stability. Eur. J. Pharm. Sci..

[10] Rathore A.S., Reason A.J. (2014). Defining Critical Quality Attributes for Monoclonal Antibody Therapeutic Products. Biopharm Int..

[11] Beck A., Nowak C., Meshulam D., Reynolds K., Chen D., Pacardo D.B., Nicholls S.B., Carven G.J., Gu Z., Fang J. (2022). Risk-Based Control Strategies of Recombinant Monoclonal Antibody Charge Variants. Antibodies.

[12] Wagner E., Colas O., Chenu S., Goyon A., Murisier A., Cianferani S., François Y., Fekete S., Guillarme D., D’aTri V. (2020). Determination of size variants by CE-SDS for approved therapeutic antibodies: Key implications of subclasses and light chain specificities. J. Pharm. Biomed. Anal..

[13] Beyer B., Schuster M., Jungbauer A., Lingg N. (2018). Microheterogeneity of Recombinant Antibodies: Analytics and Functional Impact. Biotechnol. J..

[14] Berkowitz S.A., Engen J.R., Mazzeo J.R., Jones G.B. (2012). Analytical tools for characterizing biopharmaceuticals and the implications for biosimilars. Nat. Rev. Drug Discov..

[15] Rogers R.S., Nightlinger N.S., Livingston B., Campbell P., Bailey R., Balland A. (2015). Development of a quantitative mass spectrometry multi-attribute method for characterization, quality control testing and disposition of biologics. mAbs.

[16] Zhu Y., Lou D., Yang C., Ding R., Song M., Wang R., Lu Y., Fan Q. (2026). Development and Integrated Application of the Multi-Attribute Method (MAM) in Quality Control of Biotechnological Drugs. Biotechnol. Bioeng..

[17] Millan-Martin S., Jakes C., Carillo S., Bones J. (2023). Multi-Attribute Method (MAM) Analytical Workflow for Biotherapeutic Protein Characterization from Process Development to QC. Curr. Protoc..

[18] Millan-Martin S., Jakes C., Carillo S., Rogers R., Ren D., Bones J. (2023). Comprehensive multi-attribute method workflow for biotherapeutic characterization and current good manufacturing practices testing. Nat. Protoc..

[19] Millan-Martin S., Jakes C., Carillo S., Gallagher L., Scheffler K., Broster K., Bones J. (2024). Multi-Attribute Method (MAM): An Emerging Analytical Workflow for Biopharmaceutical Characterization, Batch Release and cGMP Purity Testing at the Peptide and Intact Protein Level. Crit. Rev. Anal. Chem..

[20] Carvalho S.B., Gomes R.A., Pfenninger A., Fischer M., Strotbek M., Isidro I.A., Tugcu N., Gomes-Alves P. (2022). Multi attribute method implementation using a High Resolution Mass Spectrometry platform: From sample preparation to batch analysis. PLoS ONE.

[21] Zhang Y., Guo J. (2017). Characterization and QC of biopharmaceuticals by MS-based ‘multi-attribute method’: Advantages and challenges. Bioanalysis.

[22] Oyugi M., Wang X., Yang X., Wu D., Rogstad S. (2023). Method validation and new peak detection for the liquid chromatography-mass spectrometry multi-attribute method. J. Pharm. Biomed. Anal..

[23] Mouchahoir T., Schiel J.E., Rogers R., Heckert A., Place B.J., Ammerman A., Li X., Robinson T., Schmidt B., Chumsae C.M. (2022). Attribute Analytics Performance Metrics from the MAM Consortium Interlaboratory Study. J. Am. Soc. Mass Spectrom..

[24] Mouchahoir T., Schiel J.E., Rogers R., Heckert A., Place B.J., Ammerman A., Li X., Robinson T., Schmidt B., Chumsae C.M. (2021). New Peak Detection Performance Metrics from the MAM Consortium Interlaboratory Study. J. Am. Soc. Mass Spectrom..

[25] Kwon O., Joung J., Park Y., Kim C.W., Hong S.H. (2017). Considerations of critical quality attributes in the analytical comparability assessment of biosimilar products. Biologicals.

[26] Shipman J., Oyugi M., Marzan T.A., Geerlof-Vidavsky I., Kirkpatrick D., Zhu H., Rasangika M., Rogstad S. (2025). A Multi-Laboratory, Multi-Platform Analysis of the Multi-Attribute Method. Pharmaceuticals.

[27] Li X., Pierson N.A., Hua X., Patel B.A., Olma M.H., Strulson C.A., Letarte S., Richardson D.D. (2023). Analytical Performance Evaluation of Identity, Quality-Attribute Monitoring and New Peak Detection in a Platform Multi-Attribute Method Using Lys-C Digestion for Characterization and Quality Control of Therapeutic Monoclonal Antibodies. J. Pharm. Sci..

[28] McCarthy D., Jing L. (2024). Considerations for Robust Implementation of the Multi-Attribute Method. Bioprocess Online. https://www.bioprocessonline.com/doc/considerations-for-robust-implementation-of-the-multi-attribute-method-0001.

[29] Li X., Rawal B., Rivera S., Letarte S., Richardson D.D. (2022). Improvements on sample preparation and peptide separation for reduced peptide mapping based multi-attribute method analysis of therapeutic monoclonal antibodies using lysyl endopeptidase digestion. J. Chromatogr. A.

[30] Pastrana B., Culyba E., Nieves S., Sazinsky S.L., Canto E.I., Noda I. (2024). Streamlined Multi-Attribute Assessment of an Array of Clinical-Stage Antibodies: Relationship Between Degradation and Stability. Appl. Spectrosc..

[31] Buckley C., Millan-Martin S., Carillo S., Fussl F., MacHale C., Bones J. (2024). Implementation of a LC-MS based multi-attribute method (MAM) and intact multi-attribute method (iMAM) workflow for the characterisation of a GLP-Fc fusion protein. Anal. Biochem..

[32] Yang F., Zhang J., Buettner A., Vosika E., Sadek M., Hao Z., Reusch D., Koenig M., Chan W., Bathke A. (2023). Mass spectrometry-based multi-attribute method in protein therapeutics product quality monitoring and quality control. mAbs.

[33] Pohl T., Gervais A., Dirksen E.H., D’Alessio V., Bechtold-Peters K., Burkitt W., Cao L., Greven S., Lennard A., Li X. (2023). Technical considerations for the implementation of the multi-attribute-method by mass spectrometry in a quality control laboratory. Eur. J. Pharm. Biopharm..

[34] Rogstad S., Yan H., Wang X., Powers D., Brorson K., Damdinsuren B., Lee S. (2019). Multi-Attribute Method for Quality Control of Therapeutic Proteins. Anal. Chem..

[35] Fan W., Li X., Long Z., Pei D., Shi X., Wang G., Guo Y., Bo T., Zhou Y., Chen T. (2023). Integrating ultra-high-performance liquid chromatography tandem mass spectrometry and imaged capillary isoelectric focusing for in-depth characterization of complex fusion proteins. Rapid Commun. Mass Spectrom..

[36] Song Y.E., Dubois H., Hoffmann M., DeRi S., Fromentin Y., Wiesner J., Pfenninger A., Clavier S., Pieper A., Duhau L. (2021). Automated mass spectrometry multi-attribute method analyses for process development and characterization of mAbs. J. Chromatogr. B.

[37] Evans A.R., Hebert A.S., Mulholland J., Lewis M.J., Hu P. (2021). ID-MAM: A Validated Identity and Multi-Attribute Monitoring Method for Commercial Release and Stability Testing of a Bispecific Antibody. Anal. Chem..

[38] Evans A.R., Mulholland J., Lewis M.J., Hu P. (2024). Targeted CQA Analytical Control Strategy for Commercial Antibody Products: Replacing Ion-exchange Chromatography Methods for Charge Heterogeneity with Multi-Attribute Monitoring. mAbs.

[39] Wang D., Nowak C., Mason B., Katiyar A., Liu H. (2020). Analytical artifacts in characterization of recombinant monoclonal antibody therapeutics. J. Pharm. Biomed. Anal..

[40] Marzilli L.A., Stephens E., Philip S., English A.M., Jason C., Rouse J.C. (2017). Minimizing Method-Induced Deamidation and Isomerization During Antibody Characterization to Ensure Optimal Understanding of Product Quality Attributes. Curr. Trends Mass Spectrom..

[41] McCarthy D. (2020). Multi-Attribute Methods Supporting Quality Assessments of Therapeutic Proteins. Genetic Engineering Biotechnology News. https://www.genengnews.com/topics/drug-discovery/multi-attribute-methods-supporting-quality-assessments-of-therapeutic-proteins/.

[42] United States Pharmacopeia (2025). General Chapter, 〈1060〉 Mass Spectrometry Based Multi-Attribute Method for Therapeutic Proteins.

[43] Gupta S., Jiskoot W., Schoneich C., Rathore A.S. (2022). Oxidation and Deamidation of Monoclonal Antibody Products: Potential Impact on Stability, Biological Activity, and Efficacy. J. Pharm. Sci..

[44] Alam M.E., Slaney T.R., Wu L., Das T.K., Kar S., Barnett G.V., Leone A., Tessier P.M. (2020). Unique Impacts of Methionine Oxidation, Tryptophan Oxidation, and Asparagine Deamidation on Antibody Stability and Aggregation. J. Pharm. Sci..

[45] Gao X., Ji J.A., Veeravalli K., Wang Y.J., Zhang T., Mcgreevy W., Zheng K., Kelley R.F., Laird M.W., Liu J. (2015). Effect of individual Fc methionine oxidation on FcRn binding: Met252 oxidation impairs FcRn binding more profoundly than Met428 oxidation. J. Pharm. Sci..

[46] Goyon A., Excoffier M., Janin-Bussat M.-C., Bobaly B., Fekete S., Guillarme D., Beck A. (2017). Determination of isoelectric points and relative charge variants of 23 therapeutic monoclonal antibodies. J. Chromatogr. B.

[47] Ambrogelly A., Gozo S., Katiyar A., Dellatore S., Kune Y., Bhat R., Sun J., Li N., Wang D., Nowak C. (2018). Analytical comparability study of recombinant monoclonal antibody therapeutics. mAbs.

[48] Kaschak T., Boyd D., Lu F., Derfus G., Kluck B., Nogal B., Emery C., Summers C., Zheng K., Bayer R. (2011). Characterization of the basic charge variants of a human IgG1: Effect of copper concentration in cell culture media. mAbs.

[49] Gupta S., Schöneich C., Rathore A.S. (2024). Assessment of change in the basic variants composition of trastuzumab during dilution in saline for administration. Euro. J. Pharm. Biopharm..

[50] Wei B.C., Berning K., Quan C., Zhang Y.H.T. (2017). Glycation of antibodies: Modification, methods and potential effects on biological functions. mAbs.

[51] Vlasak J., Ionescu R. (2011). Fragmentation of monoclonal antibodies. mAbs.

[52] Fazel R., Guan Y., Vaziri B., Krisp C., Heikaus L., Saadati A., Hidayah S.N., Gaikwad M., Schlüter H. (2019). Structural and In Vitro Functional Comparability Analysis of Altebrel, a Proposed Etanercept Biosimilar: Focus on Primary Sequence and Glycosylation. Pharmaceuticals.

[53] Montacir O., Montacir H., Springer A., Hinderlich S., Mahboudi F., Saadati A., Parr M.K. (2018). Physicochemical Characterization, Glycosylation Pattern and Biosimilarity Assessment of the Fusion Protein Etanercept. Protein J..

[54] Borza B., Szigeti M., Szekrenyes A., Hajba L., Guttman A. (2018). Glycosimilarity assessment of biotherapeutics 1: Quantitative comparison of the N-glycosylation of the innovator and a biosimilar version of etanercept. J. Pharm. Biomed. Anal..

[55] DiPaola M. (2013). Development of Biosimilars: Analysis of Etanercept Glycosylation as a Case Study. J. Bioanal. Biomed..

[56] Lis S., Sharon N. (1993). Protein Glycosylation–Structural and functional aspects. Eur. J. Biochem..

[57] Cassidy B., Bloomingdale T., Carmody J. (2025). Navigating ICH Q2(R2) compliance in analytical method validation: A gap analysis toolkit to streamline risk assessment and change management. J. Pharm. Sci..

[58] Ermer J., Ermer J., Nethercote P. (2025). ICH Q2(R2): Validation of Analytical Procedures, in Method Validation in Pharmaceutical Analysis: A Guide to Best Practice.

[59] United States Pharmacopeia (2017). General Chapter, 〈1225〉 Validation of Compendial Procedures.

[60] International Council for Harmonisation (ICH) (2024). Q14 Analytical Procedure Development: Guidance for Industry.

[61] Millan-Martin S., Jakes C., Carillo S., Bones J. (2023). Multi-attribute method (MAM) to assess analytical comparability of adalimumab biosimilars. J. Pharm. Biomed. Anal..

[62] Millán-Martín S., Jakes C., Carillo S., Sutton J., Kraegenbring J., Crone C., Scheffler K., Bones J. Multi-Attribute Method for batch-to-batch comparison of Etanercept fusion protein. Proceedings of the 69th Conference on Mass Spectrometry and Allied Topics.

