# Supplementary material for: A Practical Roadmap for Implementing the LC-MS-Based Multi-Attribute Method for Analysis of Therapeutic Proteins
Source: Pharmaceuticals (Basel). 2026 Oct 19;19(10):1648. doi: 10.3390/ph19101648 (PMC13708689; doi:10.3390/ph19101648)
Supplement: Supplementary file 1 [file pharmaceuticals-19-01648-s001.zip › pharmaceuticals-4559442-supplementary.pdf]

This Supplemental Information accompanies the manuscript *"A Practical Roadmap for Implementing the LC-MS-Based Multi-Attribute Method for Analysis of Therapeutic Proteins"* and provides supporting data in the form of tables and figures. The supplemental materials present detailed analytical results and visual summaries referenced in the main text.

Together with the main manuscript, this supplemental content supports transparency and data interpretation while maintaining focus on the practical and regulatory considerations outlined in the primary article.

Table S1: Selected Adalimumab Quality Attributes Identified by LC-MS/MS Peptide Mapping

Chain	Peptide Sequence	Modifications
Heavy Chain	EEQYNSTYR	A2G0F, A2G1F, A1G0F, A1G1F, A2G2F, A2G0, A1G0, A3G0F, M3, M4, M5, M6, M7, Unglycosylated
	DTLMISR	Oxidation (M)
	EVQLVESGGGLVQPGR	Pyroglutamate (E)
	FNWYVDGVEVHNAK	Deamidation (N), Succinimide (N), Isomerization (N)
	GFYPSDIAVEWESNGQPENNYK	Deamidation (N), Succinimide (N)
	LSCAASGFTFDDYAMHWVR	Oxidation (M)
	NSLYLQMNSLR	Oxidation (M), Deamidation (N), Succinimide (N)
	SLSLSPGK	Lys Clipping (K)
	TPEVTCVVVDVSHEDPEVK	DP Clipping
	VVSVLTVLHQDWLNGK	Deamidation (N), Succinimide (N)
	WQQGNVFSCSVMHEALHNHYTQK	Oxidation (M), Deamidation (N), Succinimide (N)
	GLEWVSAITWNSGHIDYADSVEGR	Oxidation (W), Succinimide (N)
	VSNK	Deamidation (N)
Light Chain	DIQMTQSPSSLSASVGDR	Oxidation (M)
	NYLAWYQQKPGK	Oxidation (W), Deamidation (N), Succinimide (N)

Note: Highlighted amino acids indicate the sites of the corresponding PTMs.

Table S2: Selected Etanercept Quality Attributes Identified by LC-MS/MS Peptide Mapping

Region	Peptide Sequence	Modifications
**TNF-α Domain**	LPAQVAFTPYAPEPGSTCR	Deamidation (Q), O-glycan (S, T)
	EYYDQTAQMCCSK	Deamidation (Q), Oxidation (M)
	EQNR	Deamidation (Q, N)
	SMAPGAVHLPQPVSTR	O-glycan (S, T)
	CSSDQVETQACTR	NH_3 loss (Q)
**Fc Domain**	WQQGNVFSCSVMHEALHNHYTQ K	Oxidation (M, W), NH3 loss (N)
	DTLMISR	Oxidation (M)
	FNWYVDGVEVHNAK	Isomerization (D), Deamidation (N), NH3 loss (N)
	EEQYNSTYR	N-Glycans (N)
	VVSVLTVLHQDWLNGK	Deamidation (N), NH3 loss (N), Oxidation (W)
	VVSVLTVLHQDWLNGKEYK	Glycation (K)
	TPEVTCVVVDVSHEDPEVK	DP Clipping
	VSNKALPAPIEK	Glycation (K)
	EEMTK	Oxidation (M)
	GFYPSDIAVEWESNGQPENNYK	Oxidation (W), Deamidation (N), NH3 loss (N)
	SLSLSPGK	C-term K-clipping (K), Amidation (P)
	VSNK	Deamidation (N)
	TTPPVLDSDGSFFLYSK	Isomerization (D)
	NQVSLTCLVK	NH_3 loss (N), Deamidation (N)
	THTCPAPELLGGPSVFLFPPKPK	O-glycan (T, S)

Note: Highlighted amino acids indicate the sites of the corresponding PTMs.

Table S3: Example System Readiness Summary Table Showing Evaluated Parameters.

No.	Attribute	Peptide	Evaluation value (Start)	Evaluation value (End)	Acceptance Criteria
1	TIC	N/A	2717442048	3229404672	1.00E+08
2	Mass Accuracy	TTPPVLDSDGSFFLYSK	-0.3537	-1.0044	≤ 5.0 ppm
3		qIVLSQSPAILSASPGE	-0.5937	-1.4709	
4		TVAAPSVFIFPPSDEQLK	-0.1132	-0.7708	
5		DSTYSLSSTLTLSK	-1.0373	-1.1996	
6		VDNALQSGNSQESVTEQDSK	-1.1685	-1.4254	
7	MS Peak Area	qIVLSQSPAILSASPGE	39748182	40257698	1.00E+07
8		TPEVTcVVVDVSHEDPEVK	691880559	681479728	1.00E+08
9		TVAAPSVFIFPPSDEQLK	169893518	170714400	1.00E+07
10		DSTYSLSSTLTLSK	57338979	57192345	1.00E+07
11		VDNALQSGNSQESVTEQDSK	96831292	96131193	1.00E+07
12	Retention Time	TTPPVLDSDGSFFLYSK	40.79	41.03	between 39.5 and 41.5
13		TPEVTcVVVDVSHEDPEVK	28.17	28.49	between 27-29
14		TVAAPSVFIFPPSDEQLK	44.32	44.62	between 43.5 and 45.5
15		DSTYSLSSTLTLSK	27.31	27.73	between 26.5 and 28.5
16		VDNALQSGNSQESVTEQDSK	9.39	9.26	between 8.0 and 10.0
17	%Oxidation	DTLM[Ox]ISR	2.67	2.56	< 5.0%

Note: "q" denotes pyroglutamate (Pyro-Glu). "c" denotes carbamidomethylated cysteine.

Table S4: **Precision Results for Adalimumab Product Quality Attributes (PQAs).**

**PQA**	**Average %PTM**	**%RSD (*n* = 6)**
SLSLSPGK (Lys clipping)	64.98	1.00
VSNK (Deamidation)	2.52	10.14
EEQYNSTYR (A2G0F)	64.52	0.87
EEQYNSTYR (A2G1F)	15.31	1.13
EEQYNSTYR (A1G0F)	6.30	3.47
DTLMISR (Oxidation)	2.48	6.17
DIQMTQSPSSLSASVGDR (Oxidation)	0.22	5.35
NSLYLQMNSLR (Oxidation)	0.31	4.02
NSLYLQMNSLR (Deamidation)	0.22	6.43
TPEVTCVVVDVSHEDPEVK (DP Clipping)	1.53	2.61
WQQGNVFSCSVMHEALHNHYTQK (Oxidation)	0.45	2.19
EVQLVESGGGLVQPGR (Pyroglutamate)	0.58	1.69
GFYPSDIAVEWESNGQPENNYK (Deamidation)	0.84	4.69
GFYPSDIAVEWESNGQPENNYK (Succinimide)	1.28	2.52
LSCAASGFTFDDYAMHWVR (Oxidation)	1.05	3.44
GLEWVSAITWNSGHIDYADSVEGR (Oxidation)	0.51	10.41
VVSVLTVLHQDWLNGK (Succinimide)	1.14	3.12

Note: Highlighted amino acids indicate the sites of the corresponding PTMs.

Table S5: **Precision Results for Etanercept Product Quality Attributes (PQAs).**

PQAs	Day 1 (*n* = 6)		Day 2 (*n* = 6)		Overall (*n* = 12)	
	Average	%RSD	Average	%RSD	Average	%RSD
EYYDQTAQMCCSK (Oxidation)	3.73	11.52	3.87	4.58	3.81	8.10
DTLMISR (Oxidation)	2.52	2.89	2.89	5.25	2.71	8.16
WQQGNVFSCSVMHEALHNHYTQK (Oxidation)	0.69	5.21	0.74	8.00	0.71	7.34
VSNK (Deamidation)	0.82	8.38	0.97	3.71	0.90	10.23
GFYPSDIAVEWESNGQPENNYK (Deamidation)	1.07	3.20	1.19	6.09	1.13	7.24
GFYPSDIAVEWESNGQPENNYK (Succinimide)	1.12	3.11	1.15	5.79	1.14	4.80
SLSLSPGK (Incomplete Lys Clipping)	25.44	0.69	25.69	1.69	25.58	1.38
C-term SLSLSPG (Pro Amidation)	0.99	1.18	1.03	3.60	1.01	3.31
EEQYNSTYR (M5)	1.58	8.98	1.56	7.90	1.57	8.09
EEQYNSTYR (A2G2F)	6.06	2.31	5.96	5.51	6.01	4.11
EEQYNSTYR (A2G1F)	36.03	2.19	36.28	3.12	36.16	2.60
EEQYNSTYR (A2G0)	8.27	3.04	8.36	4.50	8.31	3.72
EEQYNSTYR(A2G0F)	36.88	2.04	36.72	3.69	36.80	2.85

Note: Highlighted amino acids indicate the sites of the corresponding PTMs.

Table S6: Robustness Assessment of Freeze-Thaw Stability of Adalimumab Digest Samples.

PQA	Fresh	1FT	2FT	3FT	Average	%RSD
SLSLSPGK (Lys Clipping)	65.01	63.89	66.07	65.45	65.10	1.42
DTLMISR (Oxidation)	2.74	2.57	2.66	2.64	2.65	2.67
EEQYNSTYR (A2G0F)	65.29	64.09	64.22	64.44	64.51	0.84
EEQYNSTYR (Unglycosylated)	6.00	6.29	6.24	6.30	6.21	2.25
EVQLVESGGGLVQPGR (Pyroglutamate)	0.59	0.55	0.53	0.54	0.55	5.17
NSLYLQMNSLR (Oxidation)	0.30	0.30	0.28	0.29	0.29	3.85
NSLYLQMNSLR (Deamidation)	0.21	0.13	0.13	0.13	0.15	28.74
NSLYLQMNSLR (Succinimide)	0.07	0.09	0.09	0.09	0.08	10.68
FNWYVDGVEVHNAK (Succinimide)	0.05	0.05	0.05	0.05	0.05	2.89
WQQGNVFSCSVMHEALHNHYTQK (Oxidation)	0.45	0.46	0.48	0.55	0.48	9.15
LSCAASGFTFDDYAMHWVR (Oxidation)	1.08	1.03	0.94	0.99	1.01	5.65

Note: Highlighted amino acids indicate the sites of the corresponding PTMs.

Table S7: Robustness Results from the Autosampler Stability Test of Adalimumab Digest Samples with Reinjection after 65 hours

PQA	Initial Injection	Reinjection	Average	%Difference
SLSLSPGK (Lys Clipping)	35.78	35.25	35.51	1.05
DTLMISR (Oxidation)	2.98	3.01	2.99	0.72
EEQYNSTYR(A2G0F)	64.75	64.20	64.47	0.61
EEQYNSTYR (Unglycosylated)	6.48	6.75	6.61	2.99
EVQLVESGGGLVQPGR (Pyroglutamate)	0.51	0.54	0.53	3.98
NSLYLQM[Ox]NSLR (Oxidation)	0.42	0.56	0.49	20.12
NSLYLQMNSLR (Deamidation)	0.15	0.15	0.15	0.60
NSLYLQMNSLR (Succinimide)	0.09	0.08	0.08	8.45
FNWYVDGVEVHNAK(Succinimide)	0.05	0.05	0.05	1.91
WQQGNVFSCSVMHEALHNHYTQK (Oxidation)	0.55	0.61	0.58	7.39
LSCAASGFTFDDYAMHWVR (Oxidation)	1.42	1.45	1.43	1.47

Note: Highlighted amino acids indicate the sites of the corresponding PTMs.

Table S8: New Peak Species Detected in PRTC-Spiked Humira Digest Samples

Peak ID	Reference RT (min)	Spike RT (min)	Reference m/z	Spike m/z	Control Mono Mass Exp	Mono Mass Exp	Charge State	MS Area Ratio	Peptide*
1	8.11	8.11	493.768	493.7675	985.5206	985.5206	2	n.a.	SSAAPPPPP**R**
2	8.29	8.31	613.316	613.3156	1224.6167	1224.6167	2	n.a.	GISNEGQNASI**K**
3	9.13	9.20	496.286	496.2859	990.5572	990.5572	2	n.a.	HVLTSIGE**K**
4	10.99	11.02	422.736	422.7357	843.4568	843.4568	2	n.a.	IGDYAGI**K**
5	11.42	11.46	337.228	337.2275	672.4405	672.4405	2	n.a.	PVPKP**K**
6	11.42	11.46	451.283	451.2828	900.5510	900.5510	2	n.a.	DIPVPKP**K**
7	11.42	11.46	301.191	301.1909	900.5510	900.5510	3	n.a.	DIPVPKP**K**
8	13.39	13.47	695.832	695.8318	1389.6490	1389.6490	2	n.a.	TASEFDSAIAQD**K**
9	16.14	16.21	586.799	586.7994	1171.5842	1171.5842	2	n.a.	SAAGAFGPELS**R**
10	19.25	19.37	773.895	773.8947	1545.7748	1545.7748	2	n.a.	ELGQSGVDTYLQT**K**
11	26.75	26.85	558.325	558.3251	1114.6356	1114.6356	2	n.a.	GLILVGGYGT**R**
12	27.57	27.69	745.392	745.3919	1488.7693	1488.7693	2	n.a.	SFANQPLEVVYS**K**
13	27.91	28.04	801.410	801.4099	1600.8053	1600.8053	2	n.a.	GILFVGSGVSGGEEGA**R**
14	32.15	32.21	498.802	498.8009	995.5904	995.5873	2	6882.32	LTILEEL**R**
15	36.46	36.58	573.302	573.3016	1144.5887	1144.5887	2	56774.23	NGFILDGFP**R**
16	40.11	40.27	680.373	680.3726	1358.7307	1358.7307	2	16205.82	ELASGLSFPVGF**K**
17	43.50	43.65	787.422	787.4207	1572.8301	1572.8269	2	3903.75	LSSEAPALFQFDL**K**
18	43.53	43.65	525.283	525.2827	1572.8264	1572.8264	3	1547.93	LSSEAPALFQFDL**K**

* Amino acids in bold are labeled with heavy stable isotopes. Lysine (K) is 8 Da heavier and Arginine (R) is 10 Da heavier. n.a. Not applicable.

Table S9: Summary of Average O-glycosylation Relative Abundances (%PTM) for Different Glycopeptides in Etanercept

O-glycopeptide	GalNAc-6S-3SG	GalNAc-6S	GalNAc-3SG	GalNAc-3G	GalNAc
LPAQVAFTPYAPEPGSTCR	1.94 (± 0.03)	0.01 (± 0.00)	1.82 (± 0.02)	ND	ND
SMAPGAVHLPQPVSTR	1.77 (± 0.04)	ND	85.82 (± 0.57)	5.12 (± 0.38)	4.35 (± 0.18)
THTCPAPELLGGPSVFLFPPKPK	0.75 (± 0.01)	ND	2.26 (± 0.02)	ND	0.08 (± 0.00)

Note: Values in brackets represent ±1 standard deviation (SD) from six replicate injections of three sample preparations. ND, not detected.

Table S10: Representative MAM System Readiness Criteria and Attribute-Level Transfer Acceptance Limits Used During Interlaboratory Assessment

Parameter Category	Metric/PQA Class	Representative Modality Baseline	Transfer Acceptance Limit	Rationale/Method Controls
A. System Readiness (Evaluated via USP mAb 001)	Sequence Coverage	USP mAb 001 Baseline	>95% across all runs	Verify trypsin digestion efficiency and peptide recovery at the receiving site
	Mass Accuracy (MS1)	USP mAb 001 Baseline	Within 5 ppm	Confirm MS calibration and mass stability across labs
	Retention Time Stability	USP mAb 001 Baseline	Shift ≤ 0.5 min	Ensures chromatographic alignment and peak tracking between sites
B. Product PQA Tiers	Sequence Coverage	Adalimumab	>95%	Confirms comparable digestion, LC-MS performance, and peptide identification between laboratories
		Etanercept	>85%	Reflects expected coverage limitations associated with heavily glycosylated regions while ensuring consistent performance across sites
	High Abundance (>10%) (e.g., unmodified base peptides,	Adalimumab (main forms)	Absolute difference ≤ 3.0%	Tight acceptance limit appropriate for highly abundant attributes with low analytical variability

	major N-glycans)	Etanercept (complex glycoforms/base peaks)	Absolute difference ≤ 5.0%	Accommodates increased complexity associated with glycosylated proteins
	Moderate Abundance (1-10%) (e.g., deamidation, oxidation)	Adalimumab (native deamidation/oxidation)	Absolute difference ≤ 1.5%	Reflects expected analytical variability while maintaining quantitative comparability
		Etanercept (complex glycoforms/base peaks)	Absolute difference ≤ 2.0%	Accounts for increased variability observed in heterogeneous glycoforms
	Trace-level (<1%) (e.g., glycation, Trp oxidation, rare low-level glycans)	Adalimumab (trace modifications)	Absolute difference ≤ 0.3%	Appropriate for attributes approaching lower quantitative limits
		Etanercept (low abundance O-glycans)	Absolute difference	Accommodates greater variability near the quantitation threshold

Note: Acceptance criteria were prospectively established prior to study execution and were not modified based on observed transfer outcomes. Broader acceptance limits were applied to lower-abundance attributes to account for increased relative analytical variability at low signal levels. Product-specific acceptance criteria may be refined during method validation depending on the molecule, attribute criticality, and intended application.

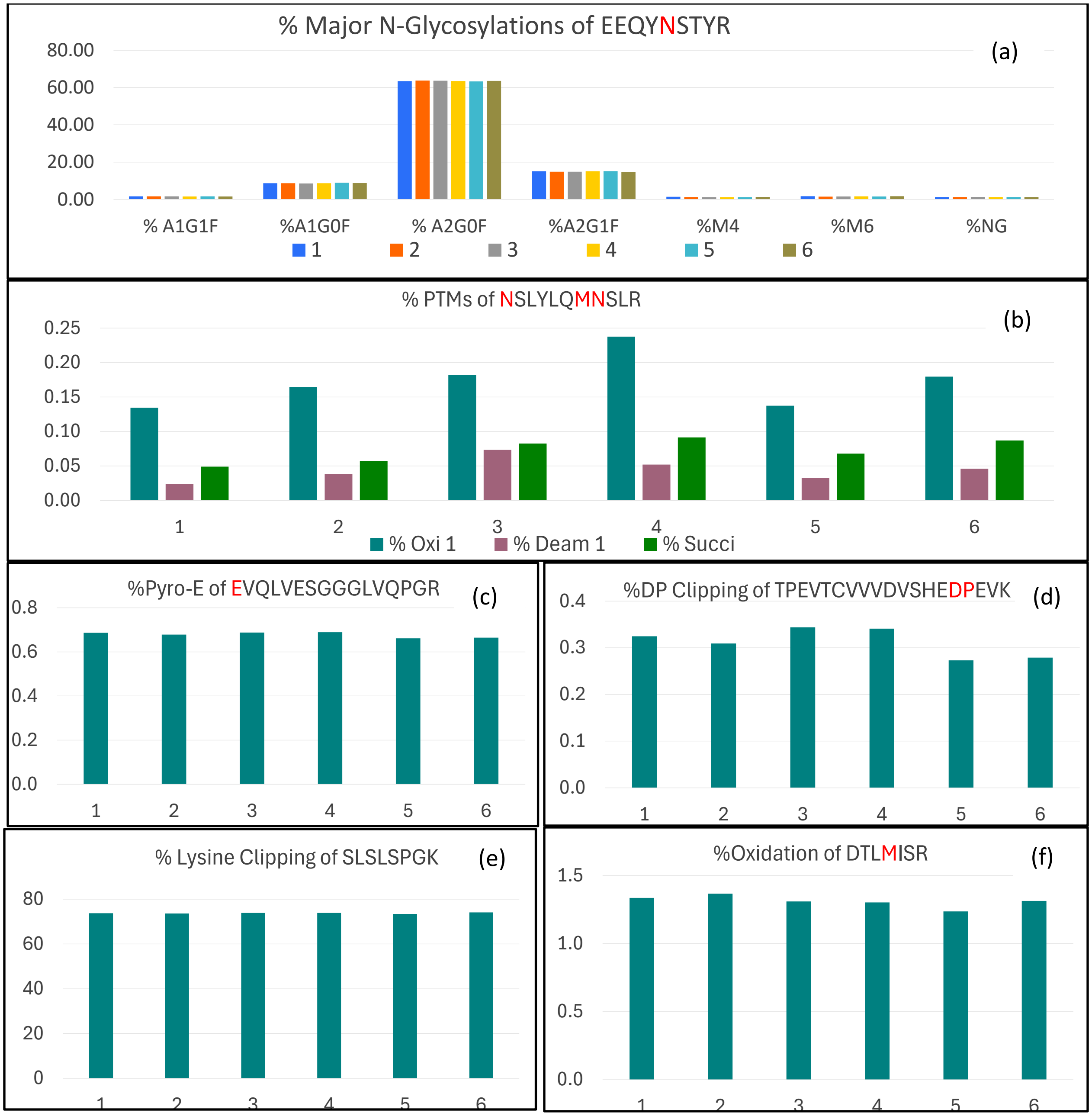

Figure S1: Relative abundance of selected adalimumab quality attributes under six sample preparation conditions. Monitored attributes include: (a) major glycoforms of EEQYNSTYR; (b) oxidation, deamidation, and succinimide of NSLYLQMNSLR; (c) N-terminal pyroglutamate of EVQLVESGGGLVQPGR; (d) DP clipping of TPEVTCVVVDVSHEDPEVK; (e) C-terminal lys clipping of SLSLSPGK; and (f) oxidation of DTLMISR. Conditions (shown on x-axis): (1) Denaturation at room temperature (RT), digestion using sequencing-grade trypsin for 30 min; (2) Denaturation at 37°C, digestion using sequencing-grade trypsin for 30 min; (3) Denaturation at RT, digestion using sequencing-grade trypsin for 60 min; (4) Denaturation at 37°C, digestion using sequencing-grade trypsin for 60 min; (5) Denaturation at RT, digestion using platinum MS-grade trypsin for 60 min; (6) Denaturation at 37°C, digestion using platinum MS-grade trypsin for 60 min.

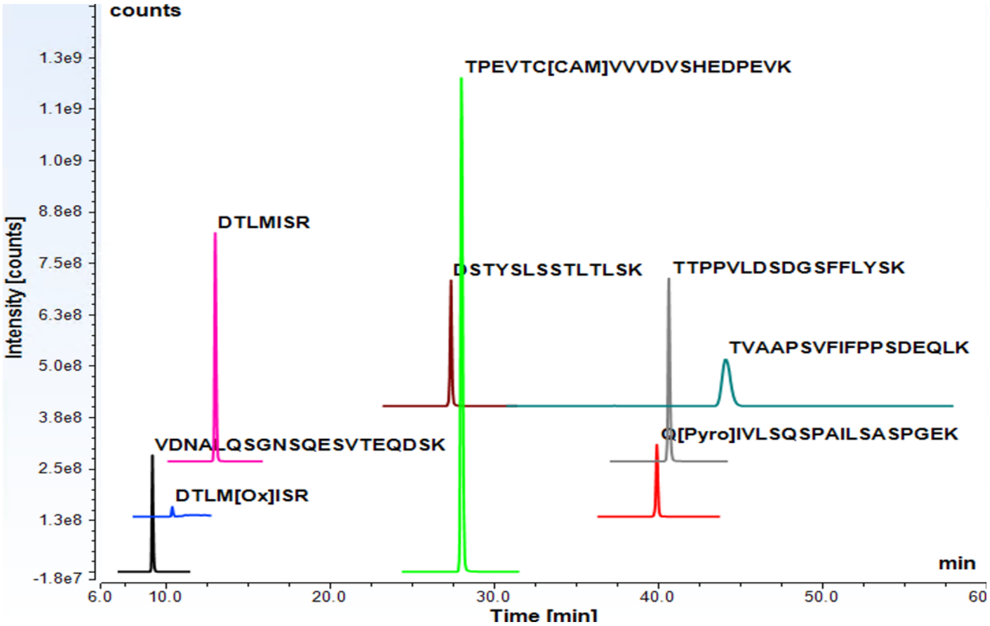

Figure S2: Chromatogram showing the elution profile of system readiness peptides, with retention time evenly distributed across the chromatographic gradient. Individual peptide traces are shown in different colors for visual distinction. The oxidized DTLMISR peptide elutes before the unmodified form during reversed-phase separation. See Table S3 for complete peptide sequences. This figure was generated using Chromeleon software (Version 7.3.2; Thermo Scientific).

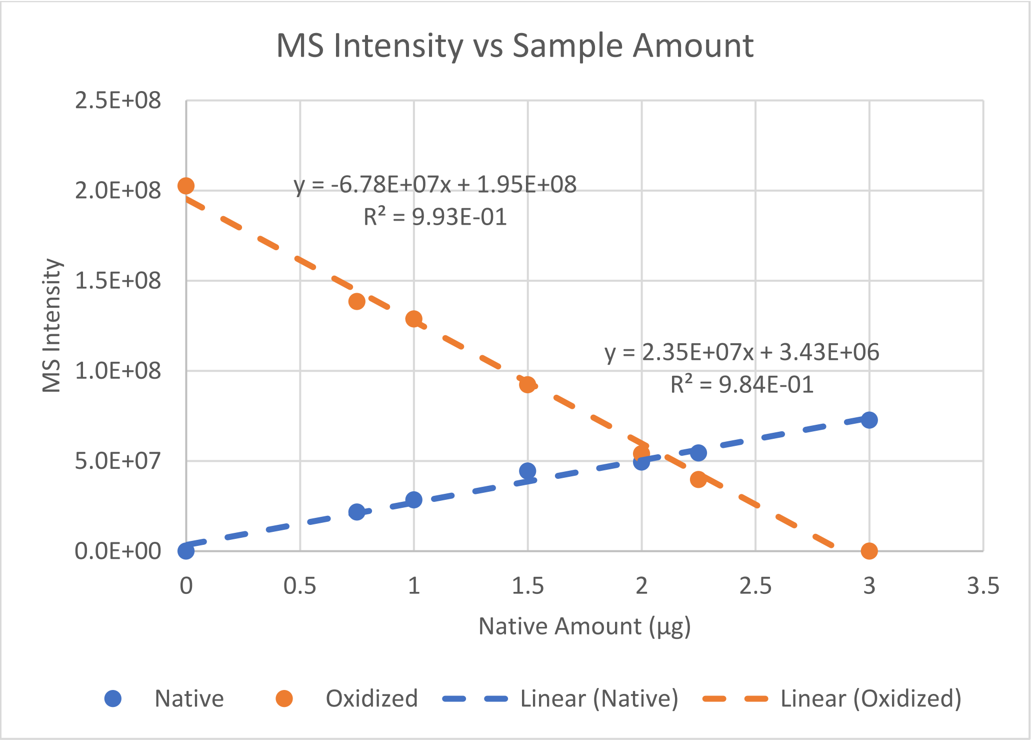

Figure S3: Linearity assessment of native (blue) and oxidized (orange) DIQMTQSPSSLSASVGDR based on injections containing 0–3 µg adalimumab.

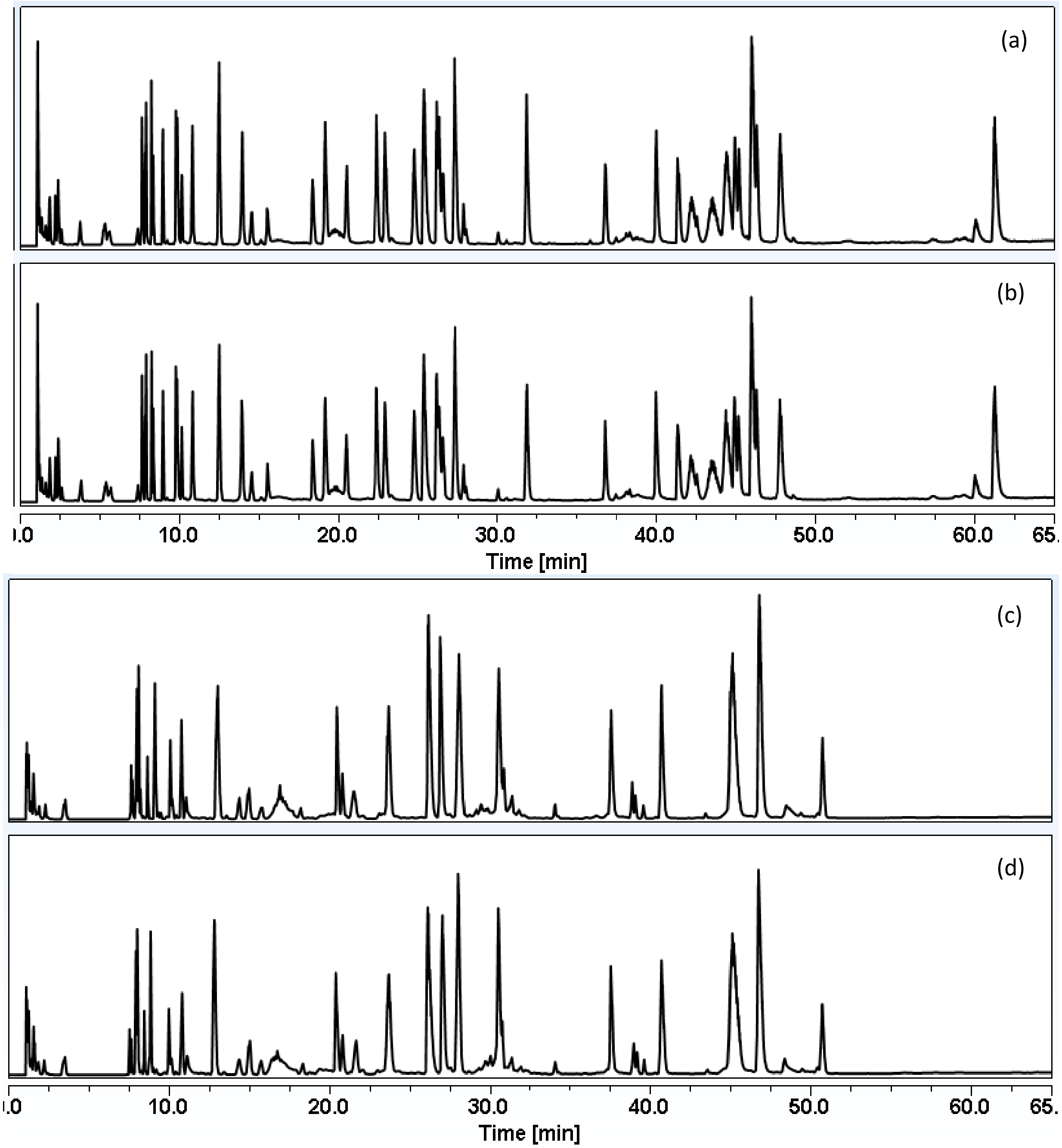

Figure S4: Total ion chromatograms (TICs) from replicate analyses of adalimumab (top panels (a) and (b)) and etanercept (bottom panels (c) and (d)). The close overlap of chromatographic profiles indicates consistent LC–MS performance across replicate samples for each biotherapeutic. This figure was generated using Xcalibur (Version 4.7.102.25; Thermo Scientific).

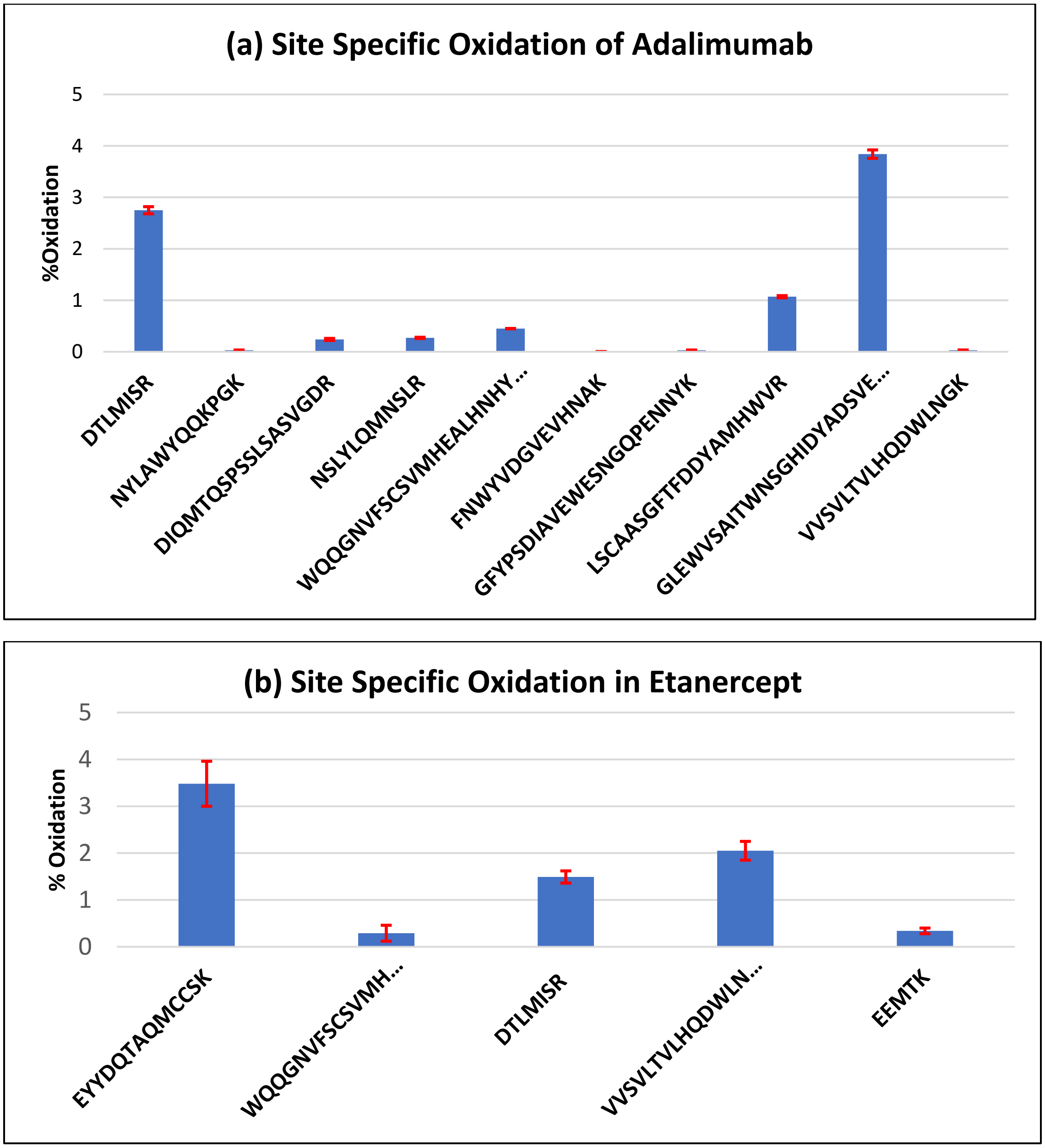

Figure S5: Peptide-level oxidation susceptibility profiles of adalimumab (a) and etanercept (b), highlighting differences in oxidation-prone regions across the two therapeutic proteins. Error bars represent ±1 standard deviation from four replicate injections of two sample preparations.

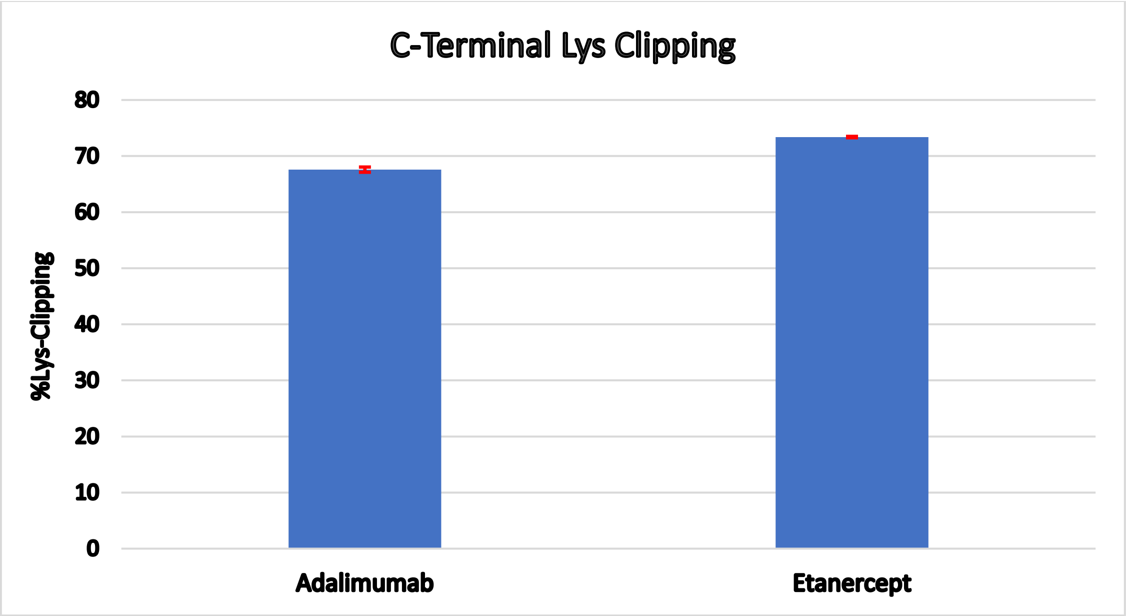

Figure S6: Relative abundance profiles of C-terminal modifications in adalimumab and etanercept measured by MAM. The low variability across replicates indicates reproducible quantitation of C-terminal attributes. Error bars represent ±1 standard deviation from four replicate injections of two sample preparations.

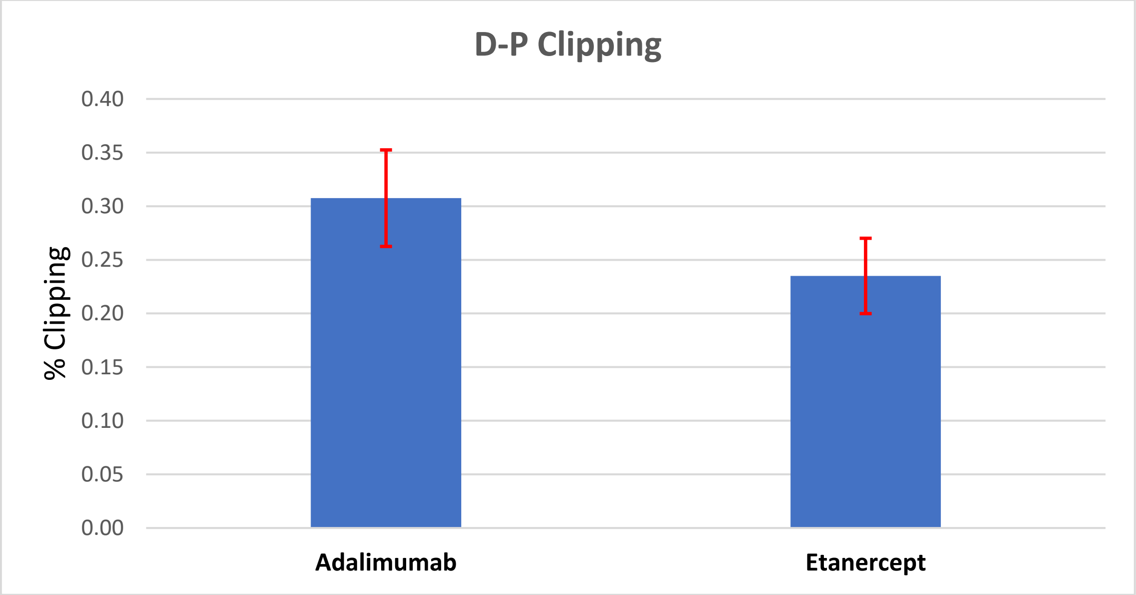

Figure S7: Relative abundance profiles of DP clipping in adalimumab and etanercept quantified by MAM, demonstrating consistent measurement of DP clipping across both therapeutic proteins. Error bars represent ±1 standard deviation from four replicate injections of two sample preparations.

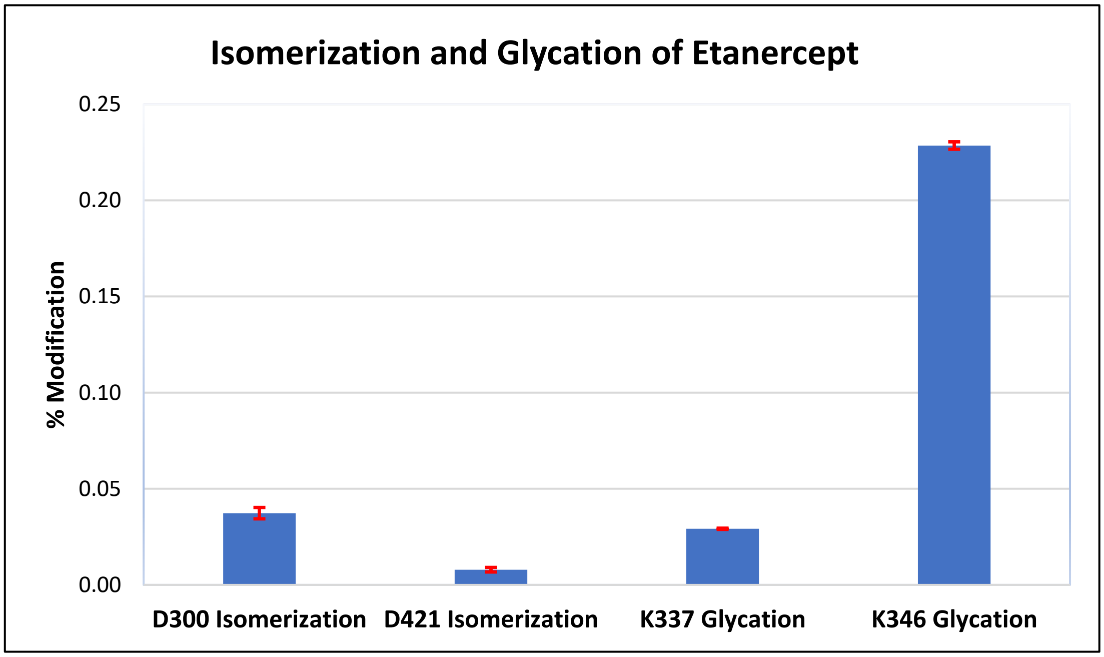

Figure S8: Mean relative percent modification for each monitored peptide in etanercept. Values were calculated from four replicate injections of two sample preparations, with error bars representing ±1 standard deviation. Isomerization and glycation levels were quantified using MAM.
